# Co-creating a person-centered creative engagement intervention for Parkinson's care

**DOI:** 10.3389/fpsyg.2024.1469120

**Published:** 2025-01-15

**Authors:** Blanca T. M. Spee, Thieme B. Stap, Marjoke Plijnaer, Gert Pasman, Sara Zeggio, Annelien Duits, Julia S. Crone, Suzanne Haeyen, Matthew Pelowski, Bastiaan R. Bloem, Jan-Jurjen Koksma

**Affiliations:** ^1^Department of Neurology, Radboud University Medical Centre, Donders Institute for Brain, Cognition and Behaviour, Centre of Expertise for Parkinson and Movement Disorders, Nijmegen, Netherlands; ^2^Vienna Cognitive Science Hub, University of Vienna, Vienna, Austria; ^3^Department of Cognition, Emotion, and Methods in Psychology, Faculty of Psychology, University of Vienna, Vienna, Austria; ^4^Radboud University Medical Center Health Academy, Nijmegen, Netherlands; ^5^Research Group Professional Workplaces, Fontys University of Applied Sciences, Eindhoven, Netherlands; ^6^Art Unbound, Collaboration Partner of Radboud University Medical Center, Nijmegen, Netherlands; ^7^Faculty of Industrial Design Engineering, Delft University of Technology, Delft, Netherlands; ^8^Department of Medical Psychology, Radboud University Medical Center, Nijmegen, Netherlands; ^9^Department of Medical Psychology, Maastricht University Medical Center, Maastricht, Netherlands; ^10^GGNet, Center for Mental Health, Scelta, Centre of Expertise for Personality Disorders, Apeldoorn, Warnsveld, Netherlands; ^11^Research Group Arts and Psychomotor Therapies in Health Care, Academy of Health and Vitality, HAN University of Applied Sciences, Nijmegen, Netherlands

**Keywords:** Parkinson's disease, creative arts therapy, transformative learning, co-creation, critical neuroscience, arts-based methods, participatory action research, creativity

## Abstract

**Background:**

Recent research in the field of “Arts and Health” has demonstrated the beneficial impact of arts-based interventions on health and well-being across diverse populations. Recognizing their potential, especially in cases where conventional healthcare cannot address the multifaceted impact of conditions such as in Parkinson's disease (PD), our study advocates for an integrative approach in medical practice and neuroscience. We recommend incorporating learning environments from the design phase through long-term care. The arts offer a unique opportunity to create such environments. In this study, we specifically focus on individuals with PD, co-designing an intervention as a creative engagement learning environment and a PD-specific creative arts therapy. In this study, the narratives of those affected contribute as scientific knowledge, shaping care and increasing the intervention's relevance to participants' lives.

**Methods:**

We used a participatory design-based research approach. Fourteen individuals with PD, along with three creative therapists and three researchers, collaborated through iterative design cycles to co-develop a creative arts therapy intervention. Qualitative data were collected through interviews, group reflections, and ethnographic observations. Data were analyzed using reflexive thematic analysis.

**Results:**

The co-creation process resulted in a 10-week creative engagement intervention delivered in a “creative playground” setting. Participants chose from multiple media and autonomously decided their creative activities. Guidance from the creative therapists was provided as needed to support individual engagement and guide reflection and learning processes. Narratives offered insights into the relevance of autonomy in care, the role of the arts, and the individuality of disease experience, resulting in seven key features of our intervention framework, which include (i) intervention structure (e.g., duration of the intervention and sessions), (ii) freedom in selection of creative media, (iii) environment as a creative playground, (iv) skills of creative therapists, (v) PD-specific considerations, (vi) financial considerations and logistics, and we list (vii) responsibilities of the Design Team.

**Discussion:**

This study establishes an initial framework for a PD-specific creative arts therapy intervention designed as a creative engagement learning environment. Future research will focus on rigorously evaluating its effectiveness and exploring its scalability in diverse settings.

## 1 Introduction

Parkinson's disease (PD) is a fast-growing neurodegenerative condition with rising prevalence worldwide (Bloem et al., 2021). As of now, 11.8 million people are diagnosed with PD (Steinmetz et al., 2024). PD is characterized by progressive motor dysfunction and a range of non-motor symptoms as well as consequences thereof—including cognitive decline, mood disturbances, and social withdrawal. Currently, healthcare for neurodegenerative diseases such as PD is delivered primarily in hospitals, offering a few, short consultations per year with hardly any daily support beyond drug treatment (Bloem et al., 2020). This traditional healthcare model incurs high costs due to its reactive nature, often leading to repeated hospital admissions and specialized treatments that could be mitigated with more proactive, integrative approaches. In addition, conventional care often struggles to adequately address the complex, multifaceted needs of people with PD, particularly in providing care that impacts their quality of life in a meaningful way within their own environments (Bloem et al., 2021). There is a growing need for interventions that increase patient empowerment and participation in care. This necessitates a shift from traditional, paternalistic healthcare models to those that support self-management and involve patients in the decision-making process (van der Scheer et al., 2017). Such interventions should recognize the responsibilities and engagement power of those affected in creating care (Göttgens and Oertelt-Prigione, 2021; Stap et al., 2023; van Woezik et al., 2023).

Creative arts therapy, as an arts-based intervention, has shown success in addressing person-centered care as a healthcare innovation (see for review, Gros et al., 2024). These interventions, led by trained creative therapists, offer therapeutic benefits through guided engagement with diverse media such as music, visual arts, and dance (Clift and Camic, 2016; Davies and Clift, 2022; Fancourt and Finn, 2019). Previous studies on people with PD, chronic diseases, and elderly populations demonstrated improvements in emotional expression and cognitive flexibility, promotion of social interaction, and well-being, with potential benefits extending to motor and non-motor symptoms (Bastepe-Gray et al., 2022; Bellass et al., 2019; de Witte et al., 2021; Feenstra et al., 2022; Groot et al., 2021; Hu et al., 2021; Maradan-Gachet et al., 2023; Pesata et al., 2022; Van Lith and Ettenberger, 2023).

To address the growing need for person-centered care in PD, partially alleviate the burden on healthcare systems, and extend the integration of creative arts therapy into PD care, we believe it is necessary to integrate individuals with PD as early as the research design stage (Göttgens and Oertelt-Prigione, 2021; McCarron et al., 2021; Mrklas et al., 2023; Vat et al., 2017; van der Scheer et al., 2017). We further propose with this integrative approach in medical practice and neuroscience to incorporate learning environments from the design phase through to long-term care (Koksma and Stap, 2023; van Woezik et al., 2023). Our goal is to move beyond static care models (Bloem et al., 2020). We aim to create dynamic environments where people with PD and practitioners co-create and evolve the therapeutic environment individually and over time. By embedding learning into the fabric of the creative intervention design, we aim to co-design an intervention that is adaptable, sustainable, and more reflective of individuals' needs while considering diverse and evolving healthcare systems (Blackburn Miller, 2020; Christie et al., 2015; Vickhoff, 2023; Willson and Jaye, 2017).

Our research is guided by two questions: (1) How can we design a creative arts therapy intervention for people with PD that is both feasible and meaningful for individuals with PD? (2) What are the key features of the established intervention that are critical for its scalability and evaluation in large-scale trials, as well as for understanding the mechanisms that contribute to its effectiveness? The outcomes of our study include the co-design process from team setup to the narratives of individuals with PD and the development of the intervention framework. We also report observed working mechanisms of the intervention and individual stories of its impact on health and well-being.

## 2 Theoretical background

Evidence-based research, reviews, and practice in the field of “Arts and Health” have consistently demonstrated the positive impact of arts-based practices on health and well-being (Fancourt and Finn, 2019; Zbranca et al., 2022). These practices encompass diverse creative domains, including music, dance, visual arts, creative writing, theater, and more (Van Lith and Ettenberger, 2023) and are broadly categorized into three forms (Davies and Clift, 2022): (1) receptive arts engagement, such as museum visits or concert attendance, often accompanied by an educational component (e.g., information about the artist or historical context); (2) open studio arts, which involve diverse, unstructured creative activities typically facilitated by an art teacher; and (3) creative arts therapies, guided by trained therapists, which aim to address cognitive, emotional, or social challenges. Our study focuses on co-designing a creative arts therapy specifically tailored for individuals with PD.

### 2.1 Parkinson's disease and the need for innovative care using the arts

PD is a complex neurodegenerative disorder that affects millions worldwide (Dorsey et al., 2018; Steinmetz et al., 2024). While motor symptoms such as tremors, bradykinesia, and rigidity are hallmark features, non-motor symptoms—including mood disturbances, cognitive inflexibility, and motivation deficits—also significantly impact individuals' quality of life (Bloem et al., 2021). These symptoms primarily result from the loss of dopamine-producing cells in the substantia nigra, a structure located in the midbrain and functionally part of the basal ganglia system. The basal ganglia system, and specifically the neurotransmitter dopamine, regulates movement, emotional responses, reward processing, and higher-level cognitive functions (Cools et al., 2022; Poewe et al., 2017; Spee et al., 2022).

Despite PD's profound effects, the brain changes are mostly invisible with traditional brain imaging techniques, and diagnosis is clinically based (Bloem et al., 2021); methods such as (functional) magnetic resonance imaging are often used for differential diagnosis of atypical degenerative variants rather than for diagnosing PD itself. As stated in the Manual of Neurological Signs (Morris and Grattan-Smith, 2015), “*Neurology is the most visual of the medical specialties.”* Clinicians often rely on observable neurological signs and how individuals use their bodies, interact with the environment, and explain their experience in daily life to infer brain function as well as treatment impact. In addition, neurological conditions manifest not as a single disorder but as a highly diverse and individual symptom spectrum, where neurobiological structures and processes intersect with societal, cultural, lifestyle, and environmental factors (Bloem et al., 2021; Bloem and Volpe, 2023).

In addition to dopamine's main functions in movement, cognition, and emotional processing, it also plays a role in creative cognition (Boot et al., 2017; Khalil et al., 2019; Lhommée et al., 2017; Spee et al., 2018). In people with PD, this relationship is visible in numerous case studies documenting artistic changes, including shifts in style, technique, and an increased creative drive (Lauring et al., 2019a,b; Pelowski et al., 2022). These artistic transformations may serve as markers for underlying brain changes associated with PD's progression and treatment (Faust-Socher et al., 2014; Inzelberg, 2013; Lhommée et al., 2014; Maradan-Gachet et al., 2023). This intersection of making art, dopamine's role in engaging in creativity activities, and PD's neurobiology has contributed to the growing recognition of arts-based interventions alongside standard PD care (Bloem and Volpe, 2023; Koksma and Stap, 2023; Pelowski et al., 2020). Managing a chronic, multifaceted condition such as PD requires innovative approaches that extend beyond conventional models (Bloem et al., 2020). Arts-based interventions, which address physical, cognitive, and emotional dimensions, offer person-centered and self-empowering solutions tailored to individual experiences and lifestyles (Osman et al., 2018; Van Lith and Ettenberger, 2023).

Studies across domains such as visual arts, music, and dance have revealed promising benefits for individuals with PD (Bastepe-Gray et al., 2022; Cucca et al., 2021; Ettinger et al., 2023; Feenstra et al., 2022; Groot et al., 2021). These interventions not only addressed motor and perceptual impairments but also showed to improve diverse aspects of quality of life, including self-efficacy, autonomy, and social connection, while reducing stigma, anxiety, and depression (Bloem et al., 2018; see for review, Gros et al., 2024). For instance, creative arts therapy using visual art was associated with increased visual-cognitive skills, emotional expression, and social interaction, leading to improved life satisfaction (Cucca et al., 2021). This structured visual art intervention, which was also used in another study (Ettinger et al., 2023), demonstrated improvements in quality of life and visual-spatial skills after 2 weekly sessions over 10 weeks. Music interventions, including randomized control trials, demonstrated benefits for motor function, speech, mood, and a reduction in stigma and anxiety with 2 weekly sessions for 6 weeks (Bastepe-Gray et al., 2022; see for review, Machado Sotomayor et al., 2021). Dance programs using a design with weekly sessions for 22 weeks for people with PD showed improvements in executive functioning, spatial awareness, and cognitive flexibility, alongside reductions in anxiety, depression, and apathy (Feenstra et al., 2022; see for review, Ismail et al., 2021; see for focus on one dance form, Rios Romenets et al., 2015).

While these studies have demonstrated encouraging outcomes, challenges in the field of Art and Health persist in transferring such interventions across diverse PD populations and settings, particularly in creating scalable and adaptable formats (Clift, 2012; Clift et al., 2021; Grebosz-Haring et al., 2022; Kaasgaard et al., 2024). Recognizing these challenges, we believe that the strength of arts-based practices lies not only in their implementation but also in their use during the design phase.

### 2.2 Designing together: co-creation, transformative learning, and participatory action research

Transformative learning forms a key theory for this study. This theory focuses on processes in which individuals fundamentally change their understanding of themselves, their condition, and their capabilities (Mezirow, 2003; Van Schalkwyk et al., 2019). Unlike traditional learning approaches, transformative learning challenges and redefines assumptions, values, and perspectives, often leading to profound personal and social change. The arts offer unique opportunities to establish transformative learning environments by fostering creativity, self-reflection, and emotional expression (Blackburn Miller, 2020; Kokkos, 2010).

Our study integrates transformative learning with arts-based approaches and participatory action research (Coghlan, 2022; Nguyen et al., 2016; Phillips et al., 2022; Willson and Jaye, 2017). Central to this is actively involving individuals with PD in the intervention's design phase. This inclusive approach shifts designing from a top-down to a collaborative one, ensuring that the intervention aligns with participants' needs and lived experiences. Participants' narratives and dialogues with healthcare professionals and researchers play a critical role in addressing epistemic injustice—the undervaluation of experiential knowledge in healthcare (Carel and Kidd, 2014). These insights might ensure that the intervention is not only scientifically grounded but also based on the realities of daily life with PD, offering care that is relevant and feasible (Florijn et al., 2023; Frank, 2005; Göttgens and Oertelt-Prigione, 2021).

Ownership is central to this co-creation process. By actively working together with individuals with PD, the model of care shifts from something provided by professionals to a dynamic process shaped collaboratively. This fosters agency, aligning the intervention with participants' values and goals, thereby enhancing its relevance and potential impact (Tsekleves et al., 2020; Voorberg et al., 2015).

To achieve this, we employ a participatory action research and a design-based approach (van der Bijl-Brouwer and Malcolm, 2020; Dorst, 2019; Peschl and Fundneider, 2014; Phillips et al., 2022; van der Bijl-Brouwer and Dorst, 2017). While using action research, participants are collaborators to the research process rather than research subjects. This helps to break traditional researcher–participant barriers, fostering an inclusive and egalitarian research environment. This approach intends to give participants a meaningful role in shaping the intervention while enriching the research process with their lived expertise. The collaborative process not only informs effective learning environments but also positions the intervention as a space for growth and mutual learning for participants and practitioners alike (Peschl and Fundneider, 2014; Woezik et al., 2021).

We followed a set of design principles that prioritized inclusion, agency, self-direction, and experiential learning (see Methods, van der Bijl-Brouwer and Malcolm, 2020; Dorst, 2019; Peschl and Fundneider, 2014; Phillips et al., 2022; van der Bijl-Brouwer and Dorst, 2017). These principles were applied through iterative processes consisting of successive cycles of creative engagement sessions, evaluation, and adjustment, enabling the intervention to adapt to participants' evolving needs and wishes. In our study, the iterative approach was implemented without predefined timelines or fixed number of iteration cycles, emphasizing flexibility and responsiveness (van der Bijl-Brouwer and Malcolm, 2020; Peschl and Fundneider, 2014).

In our study, participants' firsthand insights—including their preferences for intervention duration, number of sessions, creative media, the impact of the creative context and team dynamics, their experiences during creative processes, and their perceptions of how dopamine fluctuations affected their state, mood, and behavior—provide hands-on, phenomenological contributions to cognitive neuroscience. Our endeavor respects and amplifies the voices of individuals with PD, ensuring that their lived experiences shape the design and delivery of therapeutic environments. By bridging cognitive neuroscience with personal experiences, we aim to redefine PD treatment as inclusive, empathetic, and adaptable.

## 3 Methods

Our study employed an explorative and co-creative approach using arts-based methods (Osman et al., 2018; Willson and Jaye, 2017) and participatory action research (Phillips et al., 2022). Arts-based methods utilize the creative process as a primary mode of inquiry and engagement in research using various forms of artistic expression such as visual arts, drama, music, and dance to facilitate exploration, communication, and understanding among participants, enriching their experiential and therapeutic outcomes. Participatory action research fosters collaboration with participants actively engaging in the research design, data collection, and analysis phases. To establish a framework for co-creating a learning environment, we followed a set of design principles van der Bijl-Brouwer and Malcolm (2020):

1. **Opening up and acknowledging the interrelatedness of problems:** Recognizing the complexity and diversity of persons with PD, creative therapists, and researchers, to ensure the intervention was flexible and responsive to varied participant needs and expressions.
2. **Developing empathy within both established team members and all participants:** Fostering empathy among team members and participants to increase support and cooperation, creating a nurturing and collaborative atmosphere.
3. **Strengthening human relationships and the environment to enable learning and creativity:** We intended to co-design an adaptable, safe, and welcoming intervention space considering accessibility, comfort, and the ability to support individual creative engagement.
4. **Influence of neurological change on mental models:** We aimed to broaden understanding of PD by integrating participants' and professionals' perspectives on how putative fluctuations in dopamine levels affect cognition and behavior.
5. **Adopting an evolutionary design approach:** The intervention design was adaptive, allowing continuous refinement based on real-time feedback and evolving participant needs.

### 3.1 Participants for team assembly

We assembled a multidisciplinary team comprising researchers from fields such as neuroaesthetics, learning sciences, medicine, neuropsychology, and non-pharmacological interventions. Professional artists and creative arts therapists with extensive experience in therapeutic and co-creative practices were also included. One person with PD, trained as a researcher (patient researcher), advised us throughout the study.

### 3.2 Procedures for team assembly

During the first iteration of creative engagement, we established teams that were integral to our study's co-design processes. This initial phase was treated as a significant result of our study, reflecting the effectiveness of our collaborative approach. All team members were required to follow a set of design principles that guided the co-creation and therapeutic interactions.

### 3.3 Participants with Parkinson's disease

We included individuals diagnosed with PD, aged 18 or older, who were willing to participate, and had signed informed consent; exclusion criteria were cognitive impairment (MoCA score < 18 (Nasreddine et al., 2005).

### 3.4 Procedures: integration of persons with Parkinson's disease

Participants with PD, excluding the patient researcher, were invited through the outpatient clinic of the Radboudumc (Center of Expertise for Parkinson and Movement Disorders) and a large healthcare innovation project (Bloem et al., 2020; Ypinga et al., 2021). Prior to their participation, all participants were thoroughly informed through an information letter and discussion with a researcher about the study's objectives and procedures.

The study was conducted in accordance with the principles set forth in the Declaration of Helsinki (2013) and adhered to Good Clinical Practice guidelines. Compliance with data protection was rigorously maintained, including adherence to the General Data Protection Regulation 2018 (GDPR) and the Dutch “Algemene verordening gegevensbescherming” (AVG). Ethics approval was granted by the ethical board METC Oost-Nederland, Radboudumc (cmo-file number 2022-15919).

#### 3.4.1 Data collection methods

Our data collection methods were as follows:

- *Interviews and Group Reflections (audio and video recordings)*: Semi-structured interviews were conducted before and after the sessions. Open individual interviews and group discussions were used to explore participants' perceptions, feelings, and reflections. Interviews and group discussions were documented through audio and, in some instances, video recordings to capture detailed verbal and non-verbal interactions.
- *Ethnographical Observations*: Action researchers documented participant interactions and behaviors during creative sessions to understand the dynamics of engagement and the contextual factors influencing participation.
- *Reflective Journals*: Action researchers kept detailed journals to record their observations and reflections, providing a continuous qualitative account of the progression.
- *Photographic Documentation*: Photographs visually documented the participants' engagement, the creative outputs produced, and the overall atmosphere of the intervention settings.

### 3.5 Outcomes

Our outcomes are (1) the development of a co-designed, person-centered creative arts therapy intervention that is feasible and meaningful for individuals with PD and within PD care and (2) the identification of key features of the established intervention relevant for large-scale investigations and for deepening our understanding of potentially emerged working mechanisms in the future.

### 3.6 Qualitative data processing and analysis

Data from interviews, group discussions, and video recordings were transcribed verbatim. *In vivo* coding and reflexive thematic analysis (Braun and Clarke, 2019) were employed by three authors to systematically process this data establishing a list of codes, code clusters, and themes (Tong et al., 2007). In total, we identified 26 code clusters divided over 5 overarching themes (see Table 2 for themes and code clusters, as well as Supplementary Description D1 and Supplementary Table S1 for full list of 476 codes).

Reflexive thematic analysis is a qualitative method used to identify, analyze, and report patterns (themes) within data (see for details Braun and Clarke, 2006, 2019; Clarke and Braun, 2016). It emphasizes the active role of the researcher in interpreting data, acknowledging that themes are constructed through reflexive engagement rather than simply “emerging.” Key aspects of reflexive thematic analysis include its flexibility, the centrality of researcher reflexivity, and its iterative nature, where themes are not just data summaries but represent patterns of shared meaning underpinned by a central organizing concept. The process involves continuous reflection and re-engagement with the data during coding and theme development.

## 4 Results

Our journey toward co-creating the intervention, for and with persons with PD, started with the establishment of a Creative and a Design Team. We then report about iterations and arts-based methods applied and space configurations. In the third section, we present the narratives of individuals with PD considering “creative environment,” “being in the moment,” “making meaning,” and “people with PD about PD and dopamine.” This laid the groundwork for summarizing key features of the established intervention.

### 4.1 Design team building

We invested over 1.5 years (in total, 33 meetings, each lasting approximately 1.5 to 3 h) in assembling the right team members for the creative team and the design team.

#### 4.1.1 Formation of the creative team

From the broader team assembly, the “Creative Team” was formed, consisting of three creative arts therapists [i.e., (1) multi-media art therapist and artist with expertise in visual arts, costume creation, creative writing, drama, performance, dance, and acting; (2) music therapist and musician with expertise in digital art (visuals, film, and music); (3) dance/drama therapist]. This team focused primarily on the direct delivery of the intervention's artistic components, facilitating expressive and therapeutic activities tailored to the participants' needs. Throughout the development of the intervention, Creative Team members received training on PD-specific aspects and gained hands-on experience with this condition.

#### 4.1.2 Formation of the on-site design team

Simultaneously, the “Design Team” was established consisting of (i) three researchers (two learning researchers, one researcher from neuroaesthetics and neuropsychology) who were chosen as on-site action researchers. (ii) A member from the Creative Team was working more closely with the Design Team serving as a communicating party between the two teams. (iii) A patient-researcher provided invaluable insights from the perspective of someone living with PD but knowledgeable in scientific practices. (iv) One of the three researchers of the Design Team served as the liaison, facilitating coordination and communication among the members of the Creative and Design Team and all other researchers involved by holding regular monthly to quarterly meetings. This individual was required to be adept at bridging the diverse “languages” of research practices across both creative arts and between research disciplines.

The Design Team's role was to oversee strategic planning and integrate scientific and artistic methodologies within the intervention. They were responsible for structuring and continuously refining the learning environment, ensuring that everyone conforms to the design principles (van der Bijl-Brouwer and Malcolm, 2020), and learning/addressing the specific needs of participants with PD. During the formation of the Design Team, defining clear roles and responsibilities was crucial, which facilitated effective collaboration. We cultivated a reflective culture within the Design Team that supported ongoing reflection in-action, reflection on and over the action, and collaborative decision-making during creative engagement sessions (Tosey et al., 2012). The empathetic nature of the Design Team provided a safe and nurturing environment, fostering meaningful connections, and facilitating open communication about the disease and participants' creative experiences.

### 4.2 Creative engagement sessions: iterations and arts-based methods

We needed a total of three iterations. The first iteration was 3 h and conducted by the Design Team to bond and foster understanding within a creative engagement session context. Session was conducted in a single room in university infrastructures (school-like atmosphere). The offered art media options were visual art, music, and drama tools. Conducted activities were a short welcome, followed by drama exercises, co-creative music making, and visual arts (collages). The first iteration was finalized by an evaluation held as open discussion round.

The second and third iteration was conducted with participants with PD. See for demographic and clinical characteristics of participants with PD (Table 1).

**Table 1:** Participant with Parkinson's disease: demographics and clinical characteristics invited for second and third iteration cycle.

**Characteristics**	**Second iteration cycle**	**Third iteration cycle**
Number of participants	5	9
Age (mean and standard deviation in years)	55.5 ± 5.06	54.5 ± 9.01
Gender identity	3 women, 2 men	5 women, 4 men
Nationality	Dutch	Dutch
Educational background	Higher education	5 higher professional education, 3 secondary vocational education, 1 university degree
Living situation	With partner/family	Predominantly partnered (5 married, 2 cohabitating), 2 single and living alone
Employment status	3 working	4 incapacitated, 2 self-employed, 1 retired, 1 part-time, 1 incapacitated but active in volunteer work
Disease progression using Hoehn–Yahr (HY) scores^a^	*n* = 2, HY 1 *n* = 2, HY 2 *n* = 1, HY 3	*n* = 2, HY 1 *n* = 3, HY 2 *n* = 2, HY 3 *n* = 2, HY 4
Levodopa equivalent daily dosage (mean and standard deviation in mg)^b^	NA	814.5 ± 441.6

^a^HY scores were evaluated by using the MDS-Unified Parkinson's Disease Rating Scale (Goetz et al., 2008); ^b^drug intake from participant during second iteration cycle was not registered.

During the second iteration, we conducted two creative engagement sessions lasting 3 h conducted within 1 week. During the second iteration, our patient researcher was present as action researcher. Sessions were conducted in an art studio single room (art studio atmosphere). Initial art media options included visual art (drawing, painting, throwing colors, body painting, digital art, photography, film, and collage materials), music (guitar, various percussion instruments, piano, and digital music), drama and theater play materials, creative writing, and creative cooking (see Appendix A. Impressions second iteration). The first session started with a drama exercise played by the Creative Team. The second session started with an interactive drama/music play with all participants. In both sessions, this was followed by participants freely exploring and engaging with any creative activity they wished, with the ability to change activities at any time. A final group discussion closed each session. Feedback and insights gained were immediately integrated into planning the subsequent iteration.

Our third iteration, and second round with participants with PD, extended to a 10-week program, with weekly 90- to 120-min sessions. We used all data collection methods. Sessions were conducted in an art studio with six rooms (art studio atmosphere with possibility to pull back or work together). The same initial art media options as in the second iteration were available, with additional options including sewing and fashion, wood blocks for small constructions (similar to Lego), and more space for music creation and drama (see Appendix B. Impressions third iteration). Activities included group tuning in, initial drama/music/dance exercises (first time was conducted by Creative Team alone, all following sessions interactive play with everyone). This was followed by free exploration and engagement with creative activities. Materials were continuously adapted based on individual wishes and feasibility. A final group discussion closed each session. Adjustments and refinements were continuously made based on ongoing analysis and participant feedback.

### 4.3 Narratives: the voice from people from Parkinson's

Our first research question was how can we design a creative arts therapy intervention for people with PD that is both feasible and meaningful for individuals with PD? To answer this research questions through the perspective of the people with PD, we analyzed nine interviews (duration ranged from 1 min 56 s to 35 min 43 s) and five group discussion (duration ranged from 17 min 16 s to 69 min 35 s). Codes and themes were identified collaboratively by a team of three researchers (see Table 2 and Supplementary Table S1). To accommodate language differences of one researcher, transcripts were initially translated using AI tools (ChatGPT-3.5), after which the coding process involved merging both Dutch (2 coders) and English (1 coder) versions of the codes into themes and clusters during three rounds of 3-h discussion among the independent coders. We integrated also the results of three action researchers' ethnographical observations and reflective journals.

**Table 2:** Overview of thematic analysis (themes, code clusters, and number of codes).

**Themes**	**Theme description**	**Code clusters**	**Number of codes**
(1) Anticipation	At the outset, participants held different expectations and motivations regarding the co-design process. They expressed various assumptions about the potential value of the creativity and its connection to PD. These expectations shaped how participants engaged with the study and anticipated its impact on their personal experiences.	Imaginativeness	5
		Definitions of creativity	24
		PD and creativity	24
		Motivation and expectation	37
			90
(2) Creative Engagement	The co-design process aimed to create a learning environment—both mentally and physically—that encouraged open exploration. Participants engaged deeply through active arts engagement, exploring their convictions and taking ownership of their personal wishes and needs. This process fostered meaning making, allowing participants resonate with their own lived experiences, values, and needs.	Open ended	7
		Autonomy and freedom	7
		Sense of meaning	8
		Affective impact of release	11
		Fun and joy	13
		Flow and presence	14
		Self-expression	18
			78
(3) Design	The co-development of the intervention prioritized the needs of participants, with the creative experience being central to the process. The study supported iterative design cycles, with constant attention to how the environment and activities facilitated creativity and meaningful engagement.	Guidance	6
		Outcome creative expressions	30
		Environment design	49
			85
(4) Learning	Throughout the co-design process, participants experienced different kinds of learning.	Problem-solving	4
		Challenge	5
		Adaptivity	6
		Acceptance as creative process	15
		Learning and reflection	32
			62
(5) Living with Parkinson's	Participants reflected on their personal experiences with PD, individually recognizing their past and the disease influenced their presents and getting older. This reflection included their own interpretations of how PD affected their sense of self and self-worthiness. Participants developed personal concepts of what dopamine means and its role in their experience with the disease.	PD-peers	6
		Getting older with PD	6
		Self-worthiness	9
		Dopamine	19
		The past	21
		Hobbies	22
		Living with PD	78
			161

In the upcoming subsections, we will focus on theme (2) to (4), which describe the actual “Creative Engagement” as related to the environment design in theme “Design & Research” and associated with “Reflection and Learning.” To present these findings in a more integral way, we make use of three overarching pseudo-narrative threads: (1) the creative environment, (2) being in the present moment, (3) making meaning, and (4) people with PD about PD and dopamine.

#### 4.3.1 Creative environment

In this section, the impact of the creative environment on participants' experiences is explored. Important factors visible in the analysis are the design and atmosphere of the space, the role of guidance and support, and the overall impact of the environment on participants' engagement and well-being.

The code cluster “Environment Design” encompassed codes related to the physical and social aspects during the sessions. Participants emphasized the importance of a pleasant location and the presence of a limited number of participants, which contributed to a convivial atmosphere and a sense of community. The Design Team emphasized the openness of the intervention, while simultaneously providing structure to the session.

This fostered a sense of safety and connectedness:

“*I really liked the location, and the people who were there were great. Yes. They were kind and easy to get along with, which helped to expose yourself a bit.”*

[Dutch version: “*Deze locatie was al helemaal prettig en ok de mensen er waren super. Ja. Aardig/en gemakkelijk om mee om te gaan, om je een beetje bij bloot te geven.”*]

Participants appreciated that they did not need to explain their condition and could be themselves without judgment. Some of the individuals with PD who had participated in other research before, remarked that in those studies they did not feel like a whole person, but more like another patient adding to the data. In addition, participants in our study appreciated the group dynamics and the contribution of researchers and peers to a sense of community:

“*[...] That you have peers around you. And that during the session it goes very well, and creatively everything flows, but at other moments you are just struggling. That's not a problem because here it doesn't bring shame.”*

[Dutch version: “*Je hebt peers om je heen. En dat je de ene week of dag heel goed kunt zijn, creatief alles flowed, en het andere moment zit je gewoon te spartelen. Dat is dan geen probleem, het geeft geen schaamte.”*]

The variation and flexibility in activities were seen as essential, allowing participants to explore different creative media and techniques. Gathered from codes in the theme “Creative Engagement” (T2), the environment also allowed for autonomy and freedom, enabling participants to break free from the constraints of PD and engage in creative exploration without pressure or expectations. For this, it was crucial to help participants overcome initial hesitations and prevent them from being overwhelmed by all the creative possibilities without having assignments telling them what to do, as you would normally have in an art class for instance. It is precisely this kind of challenge that we tried to design for them, so that participants may also learn to let go and “trust the process” to take over for you, which is a mode of being they cannot enter so easily in their daily lives:

“*Look, normally, your friends do take it into account, but even with them it is difficult. So, often you set boundaries, you have to set them to protect yourself. But then you work from a negative starting point. Here, on the other hand, there is a search for what you can do and who you are, including the Parkinson's.”*

[Dutch version: “*Kijk vrienden normaal, die houden er ook wel rekening mee, maar vaak lukt dat niet. Dus, dan komt het vanuit een soort negatieve grens die je dan zelf moet aangeven. Hier wordt er juist gezocht naar wat kun je en wie ben je, inclusief de Parkinson.”*]

This way of being allowed participants to express themselves which could be experienced as truly liberating:

“*I experience Parkinson's like a lot of elastic bands around me. And then doing big things, and breaking out – that's also a bit with that painting, splash, splash – make it big, and for example... Yes, just demolishing too, yes, making it big, being able to throw everything out.”*

[Dutch version: “*Ik ervaar Parkinson als heel wat elastiekjes om mij heen. En dan dus grote dingen doen, en uitbreken – dat is ook een beetje met dat schilderen, flats, flats – groot en bijvoorbeeld… Ja, dat slopen gewoon ook, ja groot, alles eruit kunnen gooien.”*]

With respect to guidance, participants pointed out that both the facilitation by the Creative Team and the researchers' reflective questions about how they were doing supported them in overcoming challenges and enhancing their creative experiences. Particularly important was the positive angle and focus on openness and possibilities:

“*Here, the focus is really on finding out: What can you do? Who are you? Including the Parkinson's… there is room to be who you are with Parkinson's. You all respond to that very understandingly. So, that attention, that safety, it's addictive, so to speak.”*

[Dutch version: “*Hier wordt er juist gezocht naar: wat kun je? Wie ben je? Inclusief de Parkinson… hier is de ruimte om te zijn wie je bent met Parkinson. Jullie spelen daar ook heel begripvol op in. Dus die aandacht, die veiligheid, is ook verslavend, om het zo maar te zeggen.”*]

Participants recognized the paradoxical nature of their expectations, desiring both structure and freedom. They initially expressed the need to know what to expect to feel comfortable and engaged but found out that once they could reconcile themselves to “not knowing” they could regain a sense of structure in the creative activities:

“*Finding that structure again in what I can do. I had divided myself into all these compartments. I think this new structure is something like rhythm. I had it with music too, very much so… experiencing a structure that I could disappear into for a while.”*

[Dutch version: “*Die structuur weer vinden in wat ik kan doen. Ik had allemaal vakjes gemaakt. Ik denk dat dat iets van ritme is. Dat had ik met de muziek ook heel erg: een structuur om daar dan heel even in te verdwijnen.”*]

#### 4.3.2 Being in the present moment

In this section, we delve further into the participants' experiences, specifically focusing on factors of “Creative Engagement” that facilitated their ability to be fully present in the creative activities: how to get into a state of flow, the role of making fun and play, the depth of self-expression, and the influence of emotions.

Engaging in the creative activities allowed participants to enter a state of flow, where they lost track of time and became completely absorbed in the creative process. One participant stated that “*you make dopamine yourself and don't notice that, so you can enter a state of flow.”* The concept of flow describes a state of deep absorption and immersion in an activity that is both enjoyable and challenging (Csikszentmihalyi, 1990). This state is characterized by a profound sense of engagement and concentration, where time seems to disappear, and self-consciousness fades, leading to optimal performance and heightened well-being (Csikszentmihalyi and Robinson, 1990). Research in the field identified flow as the optimal experience that promotes happiness and creativity, occurring when there is a balance between the skills of the individual and the challenge of the task, an experience that we could observe during the creative engagement sessions.

Participants described the experience of flow as active, hypnotizing, and rhythmically engaging, providing a sense of joy and deep engagement, helping them to forget about time and external distractions. For getting into a flow, the activity should not be hampered by technical or conceptual difficulties. Conversely, participants also mention that getting into a flow makes the creative process itself easier. Next, there is an interesting relationship between flow, experiencing joy, and having energy, and it happened regularly that people forgot to take their medicine:

“*I notice that when I'm truly in the flow, I can work intensively for hours without feeling exhausted. It feels as if, in those moments, either more dopamine is being produced, or it's being directed somewhere. That's for you to investigate.”*

[Dutch version: “*Ik merk dat wanneer ik echt in de flow ben, ik urenlang intensief kan werken zonder me uitgeput te voelen. Het lijkt alsof je op zulke momenten ofwel meer dopamine aanmaakt, of dat het daarheen wordt gestuurd. Dat is aan jullie om te onderzoeken.”]*

Participants expressed a strong sense of enjoyment, pleasure, and happiness during the creative activities. Engaging in play was emphasized as an important aspect of the intervention. Being invited to play and witnessing the playfulness of the artists allowed participants to let go of expectations and fully immerse themselves in the creative process:

“*Nothing has to come out of it. You don't need to have a plan. At first, I thought: I want something with a dragon. But well, I couldn't get it out of my hands. That frustrates. When you just let go and try this or that, it's much more fun!”*

[Dutch version*: “Er hoeft niets te komen. Je hoeft geen plan te hebben. Ik dacht eerst: ik wil iets met een draak. Maar ja, dat ik kreeg ik niet uit mijn handen. Dat frustreert dan. Als je dat dan gewoon loslaat en gewoon dit of dat probeert, dat is veel leuker!”*]

Next, participants described the process of self-expression as satisfying and fulfilling, allowing them to convey their emotions, experiences, and innermost thoughts through their creative expressions. This depth of self-expression enabled them to explore and communicate their identity and lived experiences with PD, fostering a sense of authenticity and personal growth:

“*I find it so nice that I've rediscovered here that I also really like theater. It's a real outlet for me. Even when I'm very tired, like this morning when I came here, I forgot to take my medication last night and this morning, and then I enter here differently. And then I think, I don't know if I'll manage with drama. But then it works out so well! Yes, it really gives me energy, and I can express all my emotions in it. I leave here differently than when I arrived. More energetic. And it's also something that I'm really good at. Because in my work, I am constantly confronted with what I can no longer do. And that, of course, also affects my self-image and confidence. But this theater comes naturally to me. Switching between emotions, situations, and roles. I love acting. And I hope to carry that with me as soon as I walk out this door.”*

[Dutch version: “*Ik vind het zo fijn dat ik hier weer ontdekt heb dat ik theater ook zo leuk vind. Daar kan ik echt mijn ei in kwijt. Ook als ik heel moe ben, zoals vanmorgen toen kwam ik hier, ik heb gisteravond en vanmorgen mijn medicatie vergeten in te nemen en dan kom ik echt anders binnen hier. En dan denk ik, dat drama dat weet ik niet of dat gaat lukken. Maar dat werkt dan weer zo fijn! Ja, daar krijg ik echt energie van en ik kan er al mijn emoties in kwijt. Ik ga weer anders weg dan dat ik hier aan kwam. Energieker en het is ook iets wat ik ook heel goed kan. Want in mijn werk ik word ik iedere keer geconfronteerd met wat ik niet meer kan. En dat doet natuurlijk ook iets met mijn zelfbeeld en met mijn zelfvertrouwen. En dit theater dat gaat mij gewoon gemakkelijk af. Schakelen tussen emoties en situaties en rollen. Ik vind het zo fijn om te spelen. En dat hoop ik dan ook een beetje zodra ik deze deur uit wandel om dat mee te nemen.”*]

Emotions played a vital role in the participants' experiences during the intervention. Participants noted that art had the power to move them emotionally, allowing them to let go of fear and release emotions such as crying and anger. Theatrical exercises proved quite effective in this regard, offering the opportunity to “play with emotions” so to speak, guided by an experienced drama therapist. The affective dimension of the intervention was considered very significant as it facilitated emotional release, catharsis, and a deeper exploration of one's inner emotional landscape.

#### 4.3.3 Making meaning

In this section, we focus on factors that contribute to their general sense of meaning in living a life with PD. We specifically address the clusters and codes from the theme “Learning & Reflection.” A lot of the codes in this theme derive from the guided group reflections at the end of each session. Those reflective endings are a form of collaborative meaning making. Sharing experiences, feelings, and thoughts with one another, and listening carefully to others, helps to process these experiences and gain new insights about oneself.

The meaning making starts from the making process itself: by making music and by making paintings or photographs or poems or film, the participants already started a process of producing knowledge about themselves. We observed that the Creative Team plays a crucial role in creating the open environment that allows for this meaning making to occur at all. In doing so, the Creative Team and participants (and researchers) seize the learning opportunities which arise unbidden. During one of the sessions, for example, one of the participants, not knowing how to start, was invited to play with the percussion instruments. Hesitantly, she started to tap a tambourine. The music therapist watched her for a short while and then sat beside her to start facilitate her search for rhythm. Next, he complimented her with having a good feeling for it and asked whether she also liked to try out the piano. They played the piano for a while, and we could witness her going into the flow once she could let go and play the same melody over and over again, accompanied by the music therapist's improvisation; she found *her* rhythm, as with the percussion. Then afterward, she explained her feelings to the researcher (who came sit with her and asked, “how was that?”). Then, she told the story of her daughter playing the piano when she was little. She remembered how the music filled the place with a lively atmosphere. She told us that she and her family sold the piano when her daughter grew up. The memories all came back to her, which left her feeling grateful and a bit melancholy as well. But then again, she also felt excitement for finding out that she herself could still play, despite her condition, and the joy it had given her.

The experience and story of this participant sheds some light on the many layers of the meaning making process, and the multifactorial nature of the learning design that allows for such a process to take place. Making the experience and insights meaningful depends on what participants consider having meaning for them. Participants regularly spontaneously shared personal stories about challenges in their lives and how they see engaging creatively as having the potential to make a difference. For instance, participants highlighted the importance of adaptability and creative problem-solving in the face of the challenges posed by PD:

“*You need a certain kind of flexibility, adaptability to start doing things differently. That does require creativity.”*

[Dutch version: “*Je een bepaald soort lenigheid hebben, wendbaarheid om dingen anders te gaan doen. Dat vraagt wel creativiteit*.”]

They described how their condition requires to develop novel approaches to overcome everyday physical and practical obstacles. Engaging in the creative activities provided a platform for participants to explore alternative solutions and find out what kind of modes of being and acting helped them in doing so. During interviews conducted with participants between their creative endeavors, they explained that their ability to solve problems creatively relies on mental agility with a deeper existential dimension. In other words, it is not solely a cognitive faculty that can be boosted, as psychologists have shown in creative thinking research (Ritter and Mostert, 2017).

Engaging creatively and reflecting on their experiences helped participants to shed new light on their adaptive abilities. Facilitated by the Creative Team, they were daring enough to willingly step out of their comfort zones and embrace the challenges posed by the creative activities. In so pushing beyond their perceived limitations, they learned more about their convictions and beliefs that either held them back or empowered them to navigate and overcome obstacles. This process of perseverance and resilience fostered a sense of achievement and meaningfulness, accompanied by feelings of joy:

“*I really enjoyed doing it. That surprised me. I really thought I was going totally out of my comfort zone. I didn't feel that way, and I find that very important.”*

[Dutch version: “*Ik vond het heel erg fijn om te doen. Dat verwonderde me. Ik had echt het idee van, nu ga ik totaal uit mijn comfortzone. Dat gevoel heb ik niet gehad, en dat vind ik dan ook wel weer heel belangrijk.”*]

Another existential angle to the topic of adaptability that came up in these conversations was acceptance and letting go. Acceptance was viewed as integral to adaptability and thus part of a creative process. To them, it involves making choices, admitting failure, and letting go of old life expectations and habits. Participants recognized that accepting PD as part of their overall life journey, rather than solely as a disease, allows them to redefine their identities and find new goals. Nourishing a positive mindset was seen as an essential motor behind this creative process:

“*I do realize, and I also hear it from caregivers, that people can really find satisfaction in creative things, like dancing or making music. That your goals change in life, and that creativity and your own well-being become more central, rather than what you have to deal with in the rush of everyday life. So, creativity can actually become a beautiful new goal in your life.”*

[Dutch version: “*Ik zie toch ook wel in, en dat hoor ik ook van de zorgverleners, dat mensen echt hun bevrediging kunnen halen uit creatieve dingen, zoals dansen of muziek maken. Dat je doelen veranderen in je leven, en dat creativiteit en je eigen welbevinden centraler komen te staan, dan waar je het mee moet doen in de snelheid van het gewone leven. Dus creativiteit kan ook een mooi ander doel worden in je leven.”*]

One of the participants wrote a poem as a kind of reflective meditation to organize his thoughts in a different way (see **Box 1**, permission for publishing was received from participant and creator of poem, see Appendix C. Box B. for poem in Dutch). The poem expresses well the existential depth of the person's struggle with his condition. It also has a paradox to it, that is, while living with PD can really be hard on life, life is still appreciated to its fullest (in this case with his family around the kitchen table).

Box 1 **A poem written by a participant.**

**“*The Finiteness of Time”***

*Enjoying the small moment*.

*Here and now. Being very content*.

*For a while not that maze of thoughts*.

*What's in store for me*.

*Sometimes you can't see the forest for the trees*.

*Yet, I still give life ten out of ten*.

*Sometimes overwhelmed, often amazed*.

*How grand the space around us*.

*What is my place? What is my purpose?*

*What do I bring about?*

*What is it that I feel?*

*What remains when I am gone?*

*I don't know the unknown that I cannot know*.

*Then there is another step back, and then regret*.

*No longer being able to do what you could do before*.

*What you could do at that time*.

*But time passes without waiting*,

*for that slow man with whom we used to laugh*.

*His time is finite, that's not unique*.

*But he can do less and less…*

*If only I were not sick*.

*Enjoying the small moment*.

*Here and now. Being very content*.

Being able to appreciate what matters most in life also helps in doing away with the trivialities.

One last important asset of the creative engagement program is that it fosters openness and curiosity because participants feel that being open and curious is rewarding and leads to surprising insights and meaningful experiences. That very notion itself seems to be an important take home message for participants as well, quite literally so, because all of them make and carry out plans to organize space and time for creative engagement in their lives. For example, a participant discovered an “energy boost” from acting and expressed a wish to join a theater group, while another intended to purchase art materials to continue visual arts at home. These intentions to incorporate creative activities into their routine life indicate the intervention's potential to foster sustainable changes in the participants' lifestyles. The arts may foster the imagination of and belief in something new:

“*There is so much to discover. And if you don't try, then you won't know.”*

[Dutch version: “*Er is zoveel te ontdekken. En als je het niet probeert, dan weet je het ook niet*.”]

For the writer of the poem, the program came just-in-time during a period that he was working on improving his life by approaching it differently. He even sent us a selfie video while he was walking leisurely through nature during his holidays, whereas when we first met during the first session he was mostly in a wheelchair.

#### 4.3.4 People with Parkinson's about Parkinson's and dopamine

One prominent topic emerging under the theme “Living with PD” was the concept of “dopamine.” Participants frequently referenced dopamine when describing their lived experiences of PD, often using it as a lens through which they interpreted their condition and its fluctuating symptoms. This shared language, though rooted in subjective interpretation rather than strict scientific accuracy, provided participants with a way to articulate the impact of their condition on their daily lives.

Participants vividly described being “*acutely aware of the ebb and flow of dopamine levels,”* associating these fluctuations with (often sudden) shifts in mood, energy, less or more motor function, and their ability to engage in daily activities. One consequence of this awareness is that people with PD often attempt to maintain control by meticulously tracking time. Their daily rhythms are heavily influenced by medication schedules (often requiring multiple doses per day, with food intake adapted accordingly) and by anticipating predictable dips in energy levels. One participant reflected on the effects of medication intake:

“*When my medication wears OFF, I feel restless, as if anticipating something unnerving, which is then actually accompanied by literal tremors.”*

[Dutch version: “*Als mijn medicatie uitwerkt, voel ik me onrustig, alsof ik nerveus ben voor iets onaangenaams, plus dat ik letterlijk begin te trillen.”*]

For many, dopamine was perceived as both a biological mechanism and a metaphor for their sense of agency and well-being. Within their narratives, dopamine was portrayed as a sort of magical substance—both a neutral agent playing its role in the machinery of the brain and the essence of good emotions and vitality:

“*I think dopamine as a kind of happiness hormone or what should I call it, if that's present at a low level now, its outliers in happiness experiences are also at a low level so to speak and I think that's a bit of a flattening in myself.”*

[Dutch version: “*Ik denk dat dopamine als een soort gelukshormoon of hoe moet ik het noemen, als dat nou op een laag pitje aanwezig is, is zijn uitschieters in geluksbeleving ook op een laag pitje zeg maar en dat vind ik een beetje een vervlakking bij mezelf dat dat een beetje vervlakkend werkt.”*]

Such narratives underscore the complex interplay between the subjective experience of dopamine and its clinical management. While the word “*dopamine*” is also part of the language participants use to communicate with healthcare professionals, it is important to recognize that their understanding of the term is grounded in personal experience rather than scientific precision. Paradoxically, this may reinforce dichotomous thinking about “ON” and “OFF” states. Neurologists, for example, are often trained to define these states in terms of medication impact on symptoms, but participants' lived experiences of “ON” and “OFF” often encompass broader emotional and physical changing conditions.

Dopamine thus becomes not only a neurophysiological focus but also a framework for understanding the personal and diverse impacts of PD. Participants' insights reflect an acute awareness of how medication timing and efficacy influence their daily rhythms and how the condition manifests uniquely in their bodies, minds, and lives. For example, participants described feeling “*stuck in the freeze”* and “*caught in the energy swings,”* which made it difficult to plan their days. These experiences were often associated with feelings of lethargy and helplessness. One participant linked this experience to a “*gray blanket that comes over you”* and describes PD as an “*elusive disease.”*

Listening to these subjective accounts could help healthcare providers better align holistic care strategies with individuals' lived experiences of both medication efficacy and the habits they develop to self-manage their care (Bloem et al., 2020; Stap et al., 2023). This understanding could guide clinicians in tailoring recommendations for activities, including creative engagement, to these oscillating patterns, enabling people with PD to optimize care in ways they can control.

One intriguing possibility is aligning creative activities with the fluctuations of dopamine. Participants noted that engaging in creative tasks provided moments of relief from PD-related symptoms and challenges. Anecdotally, some suggested that engaging creatively might even “*make dopamine yourself.”* While this perception is not a neuroscientific claim, it raises important questions about the timing of creative activities in relation to the medication cycle. For example, could encouraging art activities during “OFF” states mitigate some of the physical, cognitive, or emotional symptoms experienced during these dips? Future studies might explore whether creative engagement can extend the effects of medication and, in general, care, providing additional avenues for care.

Collecting data on how people with PD conceptualize dopamine enriches our understanding of their lived experiences while bridging phenomenological insights with medical practice. By capturing how people with PD describe their condition in their own terms, we might extend the dialogue between individuals and healthcare providers, improving the alignment of treatment strategies with patients' daily realities. Furthermore, these narratives may reveal untapped opportunities for self-managed therapeutic approaches, such as leveraging creative engagement to modulate symptoms associated with dopamine fluctuations.

The implications of these narratives extend to the broader intersection of medical practice and neuroscience. Creativity, as both a process and an experience, may hold potential for influencing dopamine neurotransmission (Beaty et al., 2016; Bloem and Volpe, 2023; Boot et al., 2017; Khalil et al., 2019). While further research is needed to untangle this complex relationship, the participants' accounts suggest that creative activities could complement existing pharmacological interventions.

### 4.4 Creative engagement intervention—a 10-week program

Our second research question was, what are the key features of the established intervention that are critical for its scalability and evaluation in large-scale trials, as well as identifying mechanisms that contribute to its potential effectiveness? Above, we have discussed the creative environment out a viewpoint from the participant narratives. Here, we will go more into some of the practical considerations that relate to the code cluster “Environment Design.” Our results feeding our design choices were based on the coding but also on the observatory notes we took, and what we discussed in the weekly reflective conversations within the Design Team listening to the participants.

In total, we identified seven key features with several categories of the co-created creative engagement intervention listed in Table 3.

**Table 3:** Key features of co-created creative engagement intervention.

**Key feature**	**Category**	**Details**
**(i) Intervention Structure**	Session Structure	10-week program with sessions preferably once a week, allowing flexibility in frequency (1–2 sessions per week) to accommodate participants' availability and energy levels.
	Timing and duration	Sessions are scheduled not before 11:00 AM and not starting later than 2:00 PM, lasting ideally 90–120 min, with individual breaks for participant comfort.
	Starting	Each session begins with a 10- to 15-min opening act or interactive performance to foster tuning in and openness.
	Engagement and creativity	After opening, each participant can freely and spontaneously choose which creative activity to conduct, like in a playground, supported by a non-mandatory meta-topic for inspiration.
	Closure	Sessions conclude with a 5- to 10-min group discussion for evaluation and sharing experiences.
**(ii) Creative media**	Creative toolbox	Features a diverse range of artistic media, which is adaptable within each session and from session to session, fostering creative activities tailored to individual needs and preferences.
	Basic supplies of creative toolbox	Visual Arts: Aquarelle, oil paints, chalk, pencils, fine and rough brushes, body painting supplies, clay or sculpturing supplies, photography equipment, Polaroids, and filmmaking tools. Drama: A selection of clothes, masks, and props for role-playing. Movement and Dance: Open access to spaces and music boxes for bodily expressive activities. Creative Writing: Poetry encouraged with a variety of inspiring books, typewriters, and pen-inks. Music: Instruments available included piano, guitar, various percussion tools, a microphone for singing, and a synthesizer. Additional Crafts: Sewing, cooking, clay work, toy figures, and wood construction sets with small building blocks.
**(iii) Creative playground**	Physical layout	The Creative Playground is strategically designed with multiple rooms and versatile corners, each crafted to foster different artistic activities. This layout supports both individual exploration and collaborative projects, allowing participants to move freely based on their creative impulses.
	Ambiance and design	Each space within the playground is designed to be warm and welcoming, with aesthetic considerations that stimulate artistic expression. The environment is designed to resonate with a variety of cultural contexts, ensuring that all participants feel comfortable and inspired.
	Diverse creative opportunities	The playground is equipped to support a wide range of creative expressions, from visual arts and sculpture to drama and dance. This diversity ensures that participants can explore and engage with the medium that best suits their artistic preferences or mood at any given time.
	Functionality and accessibility	Special attention is given to making the space accessible and functional for all participants, including those with physical limitations due to PD. Adjustments and accommodations are made to ensure that everyone can participate fully in the creative process.
**(iv) Skills of creative therapists**	Expert facilitation	Creative arts therapists provide empathic professional guidance throughout the intervention. They are experts in both artistic facilitation and have training or experience working with individuals with PD, enabling them to tailor their approach to each participant's needs sensitively.
	Diverse media proficiency	Therapists are skilled across a broad spectrum of artistic media, allowing them to effectively support a wide range of creative activities. This versatility enables them to adapt to the varying interests and abilities of participants, enhancing engagement and creative expression through co-creation.
	Tailored support	The professional guidance is deliberately structured to allow therapists to manage up to four participants each. This ensures focused and personalized interaction that respects each participant's creative space and expression, fostering a collaborative and co-creative environment.
**(v) PD-specific considerations**	Addressing individual PD-challenges	The creative playground accommodates the motor, cognitive, and neuropsychological challenges that are common in PD. This includes tailoring activities and guidance to address issues such as gait problems, decision-making difficulties, and other PD-related impairments.
	Respectful environment	Careful consideration that individuals engage authentically in creative processes without the burden of constantly communicating their PD-related limitations.
	Monitoring and adaptation	Awareness of behavioral changes due to fluctuating dopamine levels and the effects of PD medications is a critical component. Creative Team monitors these aspects closely to adjust activities dynamically, ensuring that the intervention remains effective and sensitive to each participant's state throughout the session.
	Supportive amenities	Provisions such as water for medication, flexible eating times to coincide with medication schedules, easily accessible bathrooms, and calming spaces are integral to accommodating the physical needs of participants. These facilities help manage symptoms and ensure comfort, allowing participants to focus on their creative engagement and flow.
**(vi) Financial considerations and logistics**	Facility costs	Costs associated with renting or adapting spaces to suit the intervention's needs, including facility maintenance.
	Staff compensation	Salaries for creative arts therapists (including potential costs for PD-specific training), participant coordinator, and administrative staff involved in the program.
	Materials and equipment	Expenses for purchasing and maintaining a diverse array of creative materials and equipment, ensuring they are readily available and in good condition for every session.
	Accessibility measures	Provisions include travel reimbursements and special transport services to accommodate participants with significant mobility challenges, ensuring everyone can attend sessions without being tired from traveling back and forth.
	Supportive amenities	Facilities are equipped with essential amenities such as water, coffee, and snacks.
	Safety	Expenses related to creating and maintaining a safe, supportive environment, adhering to ethical guidelines, and ensuring privacy and data security.
**(vii) Responsibilities design team**	Scientific and creative integration	The Design Team ensures the intervention integrates scientific principles with creative arts therapies, maintaining a balance that supports both rigorous research and participant engagement.
	Adaptation and iteration	The team oversees the continual adaptation of the program based on feedback and emerging needs, ensuring that the intervention remains relevant and effective.
	Safety protocols	The Design Team implements and maintains rigorous health and safety protocols to ensure a safe and supportive environment. This includes regular reviews and updates of safety measures to meet the highest standards.
	Ethical compliance	Adherence to ethical guidelines is paramount. The team manages all aspects of informed consent, privacy, and data security, maintaining strict compliance to protect participant confidentiality and ensure the integrity of the intervention.
	Research integrity	The team upholds scientific standards throughout the research process, from data collection to analysis, ensuring that all methodologies meet current best practices in research ethics and methodology.
	Documentation and reporting	Accurate documentation and transparent reporting are managed to maintain the credibility of the research findings and facilitate peer review and replication studies.

## 5 Discussion

In this study, we co-designed a person-centered creative engagement intervention together with and for individuals with PD as a creative arts therapy. By grounding our research in transformative learning theory (Blackburn Miller, 2020; Kokkos, 2010; Mezirow, 2003) and using a participatory design-based approach (van der Bijl-Brouwer and Malcolm, 2020; Coghlan, 2022; Phillips et al., 2022), we aimed to not only co-create a therapeutic tool but also establish a dynamic learning environment. In this environment, individuals with PD could actively shape their care program (Clift, 2012; Fancourt and Finn, 2019; Frank, 2005; Göttgens and Oertelt-Prigione, 2021; Zbranca et al., 2022). The co-design process was our essential approach in crafting an intervention that was responsive to the lived experiences and needs of people with PD—i.e., meaningful—moving beyond traditional, static models of care within the field of chronic neurodegenerative diseases (Koksma and Stap, 2023; Koksma, 2014; Koksma and Kremer, 2019).

By embedding learning into the fabric of the intervention design, we intended to create a flexible, adaptable environment. With transformative learning as a theoretical framework, we addressed healthcare challenges in PD by increasing accessibility to care through enabling self-managed and proactive care. This approach addresses both current healthcare challenges (Bloem et al., 2020, 2021) and issues raised in the literature on arts-based interventions—namely, concerns about scalability and comparability of such interventions (Clift, 2012; Grebosz-Haring et al., 2022; Kaasgaard et al., 2024). Our approach also represents a step forward in addressing these challenges by incorporating the perspectives of “patients” from the design outset, creating a framework that we believe has the potential to be scalable and rigorously tested in the future. This approach is intended to encourage a shift from traditional care models toward a more person-centered paradigm that supports self-management and includes people with PD in decision-making processes.

We identified seven key features that form the backbone of our intervention, from session structure to creative media selection, therapist skills, and PD-specific considerations. A key feature was the creative playground, where participants could choose autonomously from multiple media. Certainly, many other arts therapies make use of a multi-media approach (Clift and Camic, 2016). Nonetheless, by co-designing the intervention together with people with PD, a “finding your own creativity approach” appears to be not only feasible for people with PD but also wished for by the participants. Moreover, both the learning environment and the creative playground highlight the need for an adaptable framework capable of responding to the high diversity and fluctuations in symptoms of PD, a flexibility that is often lacking in more traditional interventions (Araújo and Bloem, 2018; Bloem et al., 2018, 2021; Gros et al., 2024; Ypinga et al., 2021). By this, we hope to exemplify that self-managed proactive and timely healthcare delivery can be learned by those affected, when medical practice and healthcare providers allow a non-paternalistic and egalitarian innovative research and care-environment. We suggest that this integration might provide significant benefits in terms of patient autonomy and engagement, which are crucial for improving health outcomes in chronic disease management.

The narratives gathered during the sessions, particularly those around the creative environment, present-moment engagement, meaning making, and participants' perceptions of PD, reveal the depth of the learning environment we created. Participants reported entering a state of flow during the creative activities, where they became fully absorbed in the present moment, providing a temporary relief from their symptoms. This sense of flow has been shown to promote creativity (Csikszentmihalyi, 1990), and it emerged as a potential working mechanism in our intervention, particularly in relation to how participants managed their PD symptoms. Future research could further investigate this mechanism and its possible connection to dopamine functioning, which is specific to PD pathology (Bloem and Volpe, 2023; Boot et al., 2017; Khalil et al., 2019; Lauring et al., 2019a,b; Maradan-Gachet et al., 2023; Pelowski et al., 2022).

Another significant outcome of this study was the role of creative expression as a tool for meaning making. Participants engaged in creative activities that allowed them to reflect on their experiences with PD and redefine their identities in light of their condition. Group reflections in the learning environment at the end of each session reinforced this process, enabling participants to share their insights and learn from one another. This collaborative meaning making process is vital to transformative learning, which views learning as not just the acquisition of knowledge but also the transformation of one's worldview (Blackburn Miller, 2020; Christie et al., 2015; Kokkos, 2010; Koksma and Stap, 2023; Mezirow, 2003).

Participants' discussions about dopamine—even when described in metaphorical or subjective terms—highlighted how individuals with PD understand their condition and its effects on their physical, cognitive, and emotional states. This shared language, though not always scientifically valid under traditional neuroscientific terms (Koksma and Stap, 2023; Koksma, 2014), provided a way for participants to articulate their experiences (Carel and Kidd, 2014). This in turn might foster a better communication between people affected by a condition and healthcare providers. These insights offer a bridge between phenomenological experiences and scientific discourse, a connection that can deepen the understanding of how people live with chronic conditions (Carel and Kidd, 2014; Frank, 2005).

Taking a first step to rigorous empirical evaluation, we incorporated exploratively quantitative measures during the third iteration cycle to explore a list of co-primary outcomes, the results of which are detailed in a separate article (Spee et al., 2024). The analyzed co-primary outcomes were health-related quality of life, well-being, anxiety/depression, executive functioning, resilience, self-efficacy, aesthetic responsiveness, and healthcare consumption. The results showed a significant reduction in anxiety and increase in well-being. We also found slight improvements in cognitive functioning and reduced healthcare consumption.

In terms of research implications, our study contributes to the discourse on the value of design-based methodologies that prioritize user engagement and iterative development (van der Bijl-Brouwer and Malcolm, 2020). Our findings suggest that the co-design process empowered participants by giving them a direct role in shaping their care (Göttgens and Oertelt-Prigione, 2021). Empowerment through co-designing has been shown to foster greater engagement and investment in the therapeutic process (Christensen-Strynø et al., 2021; Phillips et al., 2022). Participants expressed increased confidence in continuing creative activities outside the sessions, indicating the potential long-term impact of the intervention on self-efficacy and social connectedness. However, we did not formally evaluate these outcomes in the current study, and we cannot make any claims at this stage regarding whether the co-design process itself, in addition to the intervention, empowered participants. Future research should assess the lasting effects of the established intervention on participants' health and well-being.

In response to ongoing critiques within healthcare regarding the lack of person-centered approaches, we propose several practical steps clinicians, other healthcare providers, and researchers can take to integrate our findings into practice: (1) Involve patients in the decision-making process of their treatment plans. Encourage patients to communicate their personal daily care needs and preferences, actively participating in shaping their own care. This can be supported by (2) introducing arts-based interventions as part of the treatment spectrum for PD and similar conditions. Our study co-designed a PD-specific creative arts therapy; however, arts-based methods have been broadly successful in increasing self-management and facilitating the communication of existential needs. (3) Foster an environment of collaboration across different medical specialties and near-home support to create a comprehensive person-centered treatment plan that addresses all aspects of a patient's health. This integrative approach might lead to more effective management of complex conditions such as PD. (4) Train healthcare teams and neuroscientists in transformative learning principles and arts-based methods to enrich their skills during the interactions with patients and research participants. This training encourages a holistic view of patient needs that might facilitate research and care that is meaningful beyond traditional symptom management.

Despite the success of our co-design approach, the co-designed creative engagement intervention framework must now be rigorously tested in larger-scale studies to assess its transferability and ensure effective implementation across different PD cohorts as well as creative and clinical settings.

## Data Availability

The original contributions presented in the study are included in the article/Supplementary material, further inquiries can be directed to the corresponding author.

## References

[B1] Araújo R. Bloem B. R. (2018). Listen to your patient: a fiddler's tale. Ann. Neurol. 84, 931–933. 10.1002/ana.25368 30383309

[B2] Bastepe-Gray S. Wainwright L. Lanham D. C. Gomez G. Kim J. S. Forshee Z.. (2022). GuitarPD: a randomized pilot study on the impact of nontraditional guitar instruction on functional movement and well-being in Parkinson's disease. Parkinson's Dis. 2022, 1–12. 10.1155/2022/1061045 35795456 PMC9252755

[B3] Beaty R. E. Benedek M. Silvia P. J. Schacter D. L. (2016). Creative cognition and brain network dynamics. Trends Cogn. Sci. 20, 87–95. 10.1016/j.tics.2015.10.004 26553223 PMC4724474

[B4] Bellass S. Balmer A. May V. Keady J. Buse C. Capstick A.. (2019). Broadening the debate on creativity and dementia: a critical approach. Dementia 18, 2799–2820. 10.1177/1471301218760906 29631495

[B5] Blackburn Miller J. (2020). Transformative learning and the arts: a literature review. J. Transfor. Educ. 18, 338–355. 10.1177/1541344620932877

[B6] Bloem B. R. Henderson E. J. Dorsey E. R. Okun M. S. Okubadejo N. Chan P.. (2020). Integrated and patient-centred management of Parkinson's disease: a network model for reshaping chronic neurological care. Lancet Neurol. 19, 623–634. 10.1016/S1474-4422(20)30064-8 32464101 PMC9671491

[B7] Bloem B. R. Okun M. S. Klein C. (2021). Parkinson's disease. Lancet 397, 2284–2303. 10.1016/S0140-6736(21)00218-X 33848468

[B8] Bloem B. R. Pfeijffer I. L. Krack P. (2018). Art for better health and wellbeing. BMJ. 356:k5353. 10.1136/bmj.k5353

[B9] Bloem B. R. Volpe D. (2023). What art can tell us about the human brain, in health and disease: comment on “Can we really ‘read' art to see the changing brain.” Phys. Life Rev. 45, 52–55. 10.1016/j.plrev.2023.03.002 37004337

[B10] Boot N. Baas M. van Gaal S. Cools R. De Dreu C. K. W. (2017). Creative cognition and dopaminergic modulation of fronto-striatal networks: integrative review and research agenda. Neurosci. Biobehav. Rev. 78, 13–23. 10.1016/j.neubiorev.2017.04.007 28419830

[B11] Braun V. Clarke V. (2006). Using thematic analysis in psychology. Qual. Res. Psychol. 3, 77–101. 10.1191/1478088706qp063oa32100154

[B12] Braun V. Clarke V. (2019). Reflecting on reflexive thematic analysis. Qualit. Res. Sport, Exer. Health 11, 589–597. 10.1080/2159676X.2019.1628806

[B13] Carel H. Kidd I. J. (2014). Epistemic injustice in healthcare: a philosophial analysis. Med. Health Care Philos. 17, 529–540. 10.1007/s11019-014-9560-2 24740808

[B14] Christensen-Strynø M. B. Phillips L. Frølunde L. (2021). Revitalising sensualities of ageing with Parkinson's through dance. J. Aging Stud. 59:100978. 10.1016/j.jaging.2021.100978 34794724

[B15] Christie M. Carey M. Robertson A. Grainger P. (2015). Putting transformative learning theory into practice. Austr. J. Adult Learn. 55, 9–30. 10.3316/ielapa.06907655486987834025536

[B16] Clarke V. Braun V. (2016). “Thematic analysis,” in Analysing Qualitative Data in Psychology, E. Lyons and A. Coyle (London: Sage), 84–103.

[B17] Clift S. (2012). Creative arts as a public health resource: moving from practice-based research to evidence-based practice. Perspect. Public Health 132, 120–127. 10.1177/1757913912442269 22700576

[B18] Clift S. Camic P. M. (2016). Oxford Textbook of Creative Arts, Health, and Wellbeing: International Perspectives on Practice, Policy and Research. Oxford: Oxford University Press. 10.1093/med/9780199688074.001.0001

[B19] Clift S. Phillips K. Pritchard S. (2021). The need for robust critique of research on social and health impacts of the arts. Cult. Trends 30, 442–459. 10.1080/09548963.2021.191049232894667

[B20] Coghlan D. (2022). “Action research,” in The Palgrave Encyclopedia of the Possible (Springer International Publishing), 9–16. 10.1007/978-3-030-90913-0_180

[B21] Cools R. Tichelaar J. G. Helmich R. C. G. Bloem B. R. Esselink R. A. J. Smulders K.. (2022). Role of dopamine and clinical heterogeneity in cognitive dysfunction in Parkinson's disease. Progr. Brain Res. 269, 309–343. 10.1016/bs.pbr.2022.01.012 35248200

[B22] Csikszentmihalyi M. (1990). Flow: The Psychology of Optimal Experience. New York: Harper Perennial.

[B23] Csikszentmihalyi M. Robinson R. E. (1990). The Art of Seeing: An Interpretation of the Aesthetic Encounter. Los Angeles: Getty Publications.

[B24] Cucca A. Di Rocco A. Acosta I. Beheshti M. Berberian M. Bertisch H. C.. (2021). Art therapy for Parkinson's disease. Parkins. Relat. Disor. 84, 148–154. 10.1016/j.parkreldis.2021.01.013 33526323

[B25] Davies C. R. Clift S. (2022). Arts and health glossary - a summary of definitions for use in research, policy and practice. Front. Psychol. 13:949685. 10.3389/fpsyg.2022.949685 35936315 PMC9354682

[B26] de Witte M. Orkibi H. Zarate R. Karkou V. Sajnani N. Malhotra B.. (2021). From therapeutic factors to mechanisms of change in the creative arts therapies: a scoping review. Front. Psychol. 12:678397. 10.3389/fpsyg.2021.678397 34366998 PMC8336579

[B27] Dorsey E. R. Sherer T. Okun M. S. Bloem B. R. (2018). The emerging evidence of the Parkinson pandemic. J. Parkinson's Dis. 8, S3–S8. 10.3233/JPD-181474 30584159 PMC6311367

[B28] Dorst K. (2019). Design beyond design. She Ji 5, 117–127. 10.1016/j.sheji.2019.05.001

[B29] Ettinger T. Berberian M. Acosta I. Cucca A. Feigin A. Genovese D.. (2023). Art therapy as a comprehensive complementary treatment for Parkinson's disease. Front. Hum. Neurosci. 17:1110531. 10.3389/fnhum.2023.1110531 37250693 PMC10215005

[B30] Fancourt D. Finn S. (2019). What is the Evidence on the Role of the Arts in Improving Health and Well-Being? A Scoping Review. World Health Organization. Regional Office for Europe. 32091683

[B31] Faust-Socher A. Kenett Y. N. Cohen O. S. Hassin-Baer S. Inzelberg R. (2014). Enhanced creative thinking under dopaminergic therapy in Parkinson disease. Ann. Neurol. 75, 935–942. 10.1002/ana.24181 24816898

[B32] Feenstra W. Nonnekes J. Rahimi T. Reinders-Messelink H. A. Dijkstra P. U. Bloem B. R. (2022). Dance classes improve self-esteem and quality of life in persons with Parkinson's disease. J. Neurol. 269, 5843–5847. 10.1007/s00415-022-11206-8 35763112

[B33] Florijn B. W. Kloppenborg R. Kaptein A. A. Bloem B. R. (2023). Narrative medicine pinpoints loss of autonomy and stigma in Parkinson's disease. Npj Parkinson's Dis. 9:152. 10.1038/s41531-023-00593-y 37914740 PMC10620172

[B34] Frank A. W. (2005). What is dialogical research, and why should we do it? Qual. Health Res. 15, 964–974. 10.1177/1049732305279078 16093373

[B35] Goetz C. G. Tilley B. C. Shaftman S. R. Stebbins G. T. Fahn S. Martinez-Martin P.. (2008). Movement disorder society-sponsored revision of the unified Parkinson's disease rating scale (MDS-UPDRS): scale presentation and clinimetric testing results. Movem. Disor. 23, 2129–2170. 10.1002/mds.22340 19025984

[B36] Göttgens I. Oertelt-Prigione S. (2021). The application of human-centered design approaches in health research and innovation: a narrative review of current practices. JMIR MHealth UHealth 9:e28102. 10.2196/28102 34874893 PMC8691403

[B37] Grebosz-Haring K. Thun-Hohenstein L. Schuchter-Wiegand A. K. Irons Y. Bathke A. Phillips K.. (2022). The need for robust critique of arts and health research: young people, art therapy and mental health. Front. Psychol. 13:821093. 10.3389/fpsyg.2022.821093 35222203 PMC8866174

[B38] Groot B. de Kock L. Liu Y. Dedding C. Schrijver J. Teunissen T.. (2021). The value of active arts engagement on health and well-being of older adults: a nation-wide participatory study. Int. J. Environ. Res. Public Health 18:8222. 10.3390/ijerph18158222 34360519 PMC8345976

[B39] Gros P. Spee B. T. M. Bloem B. R. Kalia L. V. (2024). If art were a drug: implications for Parkinson's disease. J. Parkinson's Dis. 14, S159–S172. 10.3233/JPD-240031 38788090 PMC11380257

[B40] Hu J. Zhang J. Hu L. Yu H. Xu J. (2021). Art therapy: a complementary treatment for mental disorders. Front. Psychol. 12:686005. 10.3389/fpsyg.2021.686005 34456801 PMC8397377

[B41] Inzelberg R. (2013). The awakening of artistic creativity and Parkinson's disease. Behav. Neurosci. 127, 256–261. 10.1037/a0031052 23316709

[B42] Ismail S. R. Lee S. W. H. Merom D. Megat Kamaruddin P. S. N. Chong M. S. Ong T.. (2021). Evidence of disease severity, cognitive and physical outcomes of dance interventions for persons with Parkinson's disease: a systematic review and meta-analysis. BMC Geriatr. 21:503. 10.1186/s12877-021-02446-w 34551722 PMC8456607

[B43] Kaasgaard M. Grebosz-Haring K. Davies C. Musgrave G. Shriraam J. McCrary J. M.. (2024). Is it premature to formulate recommendations for policy and practice, based on culture and health research? A robust critique of the CultureForHealth (2022) report. Front. Public Health 12:1414070. 10.3389/fpubh.2024.1414070 39081355 PMC11287899

[B44] Khalil R. Godde B. Karim A. A. (2019). The link between creativity, cognition, and creative drives and underlying neural mechanisms. Front. Neural Circuits 13:18. 10.3389/fncir.2019.00018 30967763 PMC6440443

[B45] Kokkos A. (2010). Transformative learning through aesthetic experience. J. Transform. Educ. 8, 155–177. 10.1177/1541344610397663

[B46] Koksma J. Stap T. B. (2023). The hare and the honey: Health care as transformative learning. Comment on “Can we really “read” art to see the changing brain?” by Pelowski et al. Phys. Life Rev. 45, 3–5. 10.1016/j.plrev.2023.02.003 36907009

[B47] Koksma J.-J. (2014). “Narrators of neuromyth,” in Innovation and responsibility: engaging with new and emerging technologies, eds. C. Coenen, A. Dijkstra, C. Fautz, J. Guivant, K. Konrad, C. Milburn, and H. van Lente (Akademische Verlagsgesellschaft), 149–163.32888311

[B48] Koksma J.-J. Kremer J. A. M. (2019). Beyond the quality illusion: the learning era. Acad. Med. 94, 166–169. 10.1097/ACM.0000000000002464 30234508

[B49] Lauring J. O. Ishizu T. Kutlikova H. H. Dörflinger F. Haugbøl S. Leder H.. (2019a). Why would Parkinson's disease lead to sudden changes in creativity, motivation, or style with visual art? A review of case evidence and new neurobiological, contextual, and genetic hypotheses. Neurosci. Biobehav. Rev. 100, 129–165. 10.1016/j.neubiorev.2018.12.016 30629980

[B50] Lauring J. O. Pelowski M. Specker E. Ishizu T. Haugbøl S. Hollunder B.. (2019b). Parkinson's disease and changes in the appreciation of art: a comparison of aesthetic and formal evaluations of paintings between PD patients and healthy controls. Brain Cogn. 136:103597. 10.1016/j.bandc.2019.103597 31491732

[B51] Lhommée E. Batir A. Quesada J.-L. Ardouin C. Fraix V. Seigneuret E.. (2014). Dopamine and the biology of creativity: lessons from Parkinson's disease. Front. Neurol. 5:55. 10.3389/fneur.2014.00055 24795692 PMC4001035

[B52] Lhommée E. Boyer F. Wack M. Pélissier P. Klinger H. Schmitt E.. (2017). Personality, dopamine, and Parkinson's disease: Insights from subthalamic stimulation. Movem. Disor. 32, 1191–1200. 10.1002/mds.27065 28643887

[B53] Machado Sotomayor M. J. Arufe-Giráldez V. Ruíz-Rico G. Navarro-Patón R. (2021). Music therapy and Parkinson's disease: a systematic review from 2015–2020. Int. J. Environ. Res. Public Health 18:11618. 10.3390/ijerph182111618 34770129 PMC8582661

[B54] Maradan-Gachet M. E. Debove I. Lhommée E. Krack P. (2023). “Creativity and Parkinson's disease,” in Illuminating the Intersection of Creativity and the Changing Brain, eds. A. Richard, M. Pelowski, and B. T. M. Spee (Humana, Cham. Springer International Publishing), 65–89. 10.1007/978-3-031-14724-1_3

[B55] McCarron T. L. Clement F. Rasiah J. Moran C. Moffat K. Gonzalez A.. (2021). Patients as partners in health research: a scoping review. Health Expect. 24, 1378–1390. 10.1111/hex.13272 34153165 PMC8369093

[B56] Mezirow J. (2003). Transformative learning as discourse. J. Transfor. Educ. 1, 58–63. 10.1177/1541344603252172

[B57] Morris J. G. Grattan-Smith P. J. (2015). Manual of Neurological Signs. New York: Oxford University Press. 10.1093/med/9780199945795.001.0001

[B58] Mrklas K. J. Boyd J. M. Shergill S. Merali S. Khan M. Nowell L.. (2023). Tools for assessing health research partnership outcomes and impacts: a systematic review. Health Res. Policy Syst. 21:3. 10.1186/s12961-022-00937-9 36604697 PMC9817421

[B59] Nasreddine Z. S. Phillips N. A. BÃ©dirian V. Charbonneau S. Whitehead V. Collin I.. (2005). The montreal cognitive assessment, MoCA: a brief screening tool for mild cognitive impairment. J. Am. Geriatr. Soc. 53, 695–699. 10.1111/j.1532-5415.2005.53221.x 15817019

[B60] Nguyen M. Miranda J. Lapum J. Donald F. (2016). Arts-based learning: a new approach to nursing education using andragogy. J. Nurs. Educ. 55, 407–410. 10.3928/01484834-20160615-10 27351611

[B61] Osman M. Eacott B. Willson S. (2018). Arts-based interventions in healthcare education. Med. Humanit. 44, 28–33. 10.1136/medhum-2017-011233 28823994

[B62] Pelowski M. Spee B. T. M. Arato J. Dörflinger F. Ishizu T. Richard A. (2022). Can we really “read” art to see the changing brain? A review and empirical assessment of clinical case reports and published artworks for systematic evidence of quality and style changes linked to damage or neurodegenerative disease. Phys. Life Rev. 43, 32–95. 10.1016/j.plrev.2022.07.005 36179555

[B63] Pelowski M. Spee B. T. M. Richard A. Krack P. Bloem B. R. (2020). What Parkinson's reveals about the artistic spark. Am. Sci. 108, 240–244. 10.1511/2020.108.4.240

[B64] Pesata V. Colverson A. Sonke J. Morgan-Daniel J. Schaefer N. Sams K.. (2022). Engaging the arts for wellbeing in the United States of America: a scoping review. Front. Psychol. 12:791773. 10.3389/fpsyg.2021.791773 35222154 PMC8863598

[B65] Peschl M. F. Fundneider T. (2014). Designing and Enabling Spaces for collaborative knowledge creation and innovation: from managing to enabling innovation as socio-epistemological technology. Comput. Human Behav. 37, 346–359. 10.1016/j.chb.2012.05.027

[B66] Phillips L. Christensen-Stryn,ø M. B. Frølunde L. (2022). Arts-based co-production in participatory research: harnessing creativity in the tension between process and product. Evid. Policy 18, 391–411. 10.1332/174426421X16445103995426

[B67] Poewe W. Seppi K. Tanner C. M. Halliday G. M. Brundin P. Volkmann J.. (2017). Parkinson disease. Nat. Rev. Dis. Primers 3:17013. 10.1038/nrdp.2017.13 28332488

[B68] Rios Romenets S. Anang J. Fereshtehnejad S.-M. Pelletier A. Postuma R. (2015). Tango for treatment of motor and non-motor manifestations in Parkinson's disease: a randomized control study. Complement. Ther. Med. 23, 175–184. 10.1016/j.ctim.2015.01.015 25847555

[B69] Ritter S. M. Mostert N. (2017). Enhancement of creative thinking skills using a cognitive-based creativity training. J. Cogn. Enhanc. 1, 243–253. 10.1007/s41465-016-0002-3

[B70] Spee B. Ishizu T. Leder H. Mikuni J. Kawabata H. Pelowski M. (2018). Neuropsychopharmacological aesthetics: a theoretical consideration of pharmacological approaches to causative brain study in aesthetics and art. Progr. Brain Res. 237, 343–372. 10.1016/bs.pbr.2018.03.021 29779743

[B71] Spee B. T. M. de Vries N. M. Zeggio S. Plijnaer M. Koksma J. J. Duits A. A.. (2024). Unleashing creativity in people with Parkinson's disease: a pilot study of a co-designed creative arts therapy. J. Neurol. 10.1007/s00415-024-12878-0 PMC11758163 39849173

[B72] Spee B. T. M. Sladky R. Fingerhut J. Laciny A. Kraus C. Carls-Diamante S.. (2022). Repeating patterns: predictive processing suggests an aesthetic learning role of the basal ganglia in repetitive stereotyped behaviors. Front. Psychol. 13:930293. 10.3389/fpsyg.2022.930293 36160532 PMC9497189

[B73] Stap T. B. Grol R. Laan R. Munneke M. Bloem B. R. Koksma J.-J. (2023). “Holding still, together: person-centered parkinson's care portrayed,” in Art and Neurological Disorders: Illuminating the Intersection of Creativity and the Changing Brain, eds. A. Richard, M. Pelowski, and B. T. M. Spee (Cham: Springer International Publishing), 197–214. 10.1007/978-3-031-14724-1_8

[B74] Steinmetz J. D. Seeher K. M. Schiess N. Nichols E. Cao B. Servili C.. (2024). Global, regional, and national burden of disorders affecting the nervous system, 1990–2021: a systematic analysis for the Global Burden of Disease Study 2021. Lancet Neurol. 23, 344–381. 10.1016/S1474-4422(24)00038-3 38493795 PMC10949203

[B75] Tong A. Sainsbury P. Craig J. (2007). Consolidated criteria for reporting qualitative research (COREQ): a 32-item checklist for interviews and focus groups. Int. J. Qual. Health Care 19, 349–357. 10.1093/intqhc/mzm042 17872937

[B76] Tosey P. Visser M. Saunders M. N. (2012). The origins and conceptualizations of ‘triple-loop' learning: A critical review. *Manag. Learn*. 43, 291–307. 10.1177/1350507611426239

[B77] Tsekleves E. Bingley A. F. Luján Escalante M. A. Gradinar A. (2020). Engaging people with dementia in designing playful and creative practices: co-design or co-creation? Dementia 19, 915–931. 10.1177/1471301218791692 30089400

[B78] van der Bijl-Brouwer M. Dorst K. (2017). Advancing the strategic impact of human-centred design. Design Stud. 53, 1–23. 10.1016/j.destud.2017.06.003

[B79] van der Bijl-Brouwer M. Malcolm B. (2020). Systemic design principles in social innovation: a study of expert practices and design rationales. She Ji 6, 386–407. 10.1016/j.sheji.2020.06.001

[B80] van der Scheer L. Garcia E. van der Laan A. L. van der Burg S. Boenink M. (2017). The benefits of patient involvement for translational research. Health Care Anal. 25, 225–241. 10.1007/s10728-014-0289-0 25537464

[B81] Van Lith T. Ettenberger M. (2023). Arts-based therapies, practices, and interventions in health. BMC Complem. Med. Ther. 23:351. 10.1186/s12906-023-04177-4 37794363 PMC10548758

[B82] Van Schalkwyk S. C. Hafler J. Brewer T. F. Maley M. A. Margolis C. McNamee L.. (2019). Transformative learning as pedagogy for the health professions: a scoping review. Med. Educ. 53, 547–558. 10.1111/medu.13804 30761602

[B83] van Woezik T. E. T. Stap T. B. van der Wilt G. J. Reuzel R. P. B. Koksma J.-J. (2023). Seeing the other: how residents expand their perspective by learning with the arts. J. Grad. Med. Educ. 15, 50–58. 10.4300/JGME-D-22-00140.1 36817544 PMC9934825

[B84] Vat L. E. Ryan D. Etchegary H. (2017). Recruiting patients as partners in health research: a qualitative descriptive study. Res. Involv. Engagem. 3:15. 10.1186/s40900-017-0067-x 29062540 PMC5611573

[B85] Vickhoff B. (2023). Why art? The role of arts in arts and health. Front. Psychol. 14:765019. 10.3389/fpsyg.2023.765019 37034911 PMC10075207

[B86] Voorberg W. H. Bekkers V. J. J. M. Tummers L. G. (2015). A systematic review of co-creation and co-production: embarking on the social innovation journey. Public Manag. Rev. 17, 1333–1357. 10.1080/14719037.2014.930505

[B87] Willson S. Jaye P. (2017). Arts-based learning for a circle of care. Lancet 390, 642–643. 10.1016/S0140-6736(17)31970-0

[B88] Woezik T. E. T. Stap T. B. Wilt G. J. Reuzel R. P. B. Koksma J. (2021). Transforming the medical perspective through the arts. Med. Educ. 55, 1324–1325. 10.1111/medu.14618 34455624 PMC9290063

[B89] Ypinga J. H. L. Van Halteren A. D. Henderson E. J. Bloem B. R. Smink A. J. Tenison E.. (2021). Rationale and design to evaluate the PRIME Parkinson care model: a prospective observational evaluation of proactive, integrated and patient-centred Parkinson care in The Netherlands (PRIME-NL). BMC Neurol. 21:286. 10.1186/s12883-021-02308-3 34294077 PMC8298196

[B90] Zbranca R. Dâmaso M. Blaga O. Kiss K. Dascal M. D. Yakobson D.. (2022). CultureForHealth Report. Culture's contribution to health and well-being. A report on evidence and policy recommendations for Europe. Available at: https://www.cultureforhealth.eu/app/uploads/2023/02/Final_C4H_FullReport_small.pdf (accessed July 23, 2024).

