# Supplementary material for: Co-creating a person-centered creative engagement intervention for Parkinson's care
Source: Front Psychol. 2025 Jan 15;15:1469120. doi: 10.3389/fpsyg.2024.1469120 (PMC11774897; doi:10.3389/fpsyg.2024.1469120)
Supplement: Supplementary file 1 [file Data_Sheet_1.pdf]

Supplementary Materials

Co-Creating a Person-Centered Creative Engagement Intervention for Parkinson's Care

Blanca T.M. Spee[1,2,3,*‡], Thieme B. Stap[4,5,‡], Marjoke Plijnaer[6], Gert Pasman[7], Sara Zeggio[1], Annelien Duits[8,9], Julia S. Crone[2], Suzanne Haeyen[10,11], Matthew Pelowski[2,3], Bastiaan R. Bloem[1], Jan-Jurjen Koksma[4]

[1] Radboud university medical center, Donders Institute for Brain, Cognition and Behaviour; Department of Neurology, Centre of Expertise for Parkinson & Movement Disorders, Nijmegen, the Netherlands

[2] Vienna Cognitive Science Hub, University of Vienna, Vienna, Austria

[3] Department of Cognition, Emotion, and Methods in Psychology, Faculty of Psychology, University of Vienna, Vienna, Austria

[4] Radboud university medical center Health Academy, Nijmegen, the Netherlands

[5] Fontys University of Applied Sciences, Research Group Professional Workplaces, Eindhoven, The Netherlands

[6] Art Unbound, collaboration partner of Radboud university medical center, Nijmegen, the Netherlands

[7] Faculty of Industrial Design Engineering, Delft University of Technology, Delft, the Netherlands

[8] Department of Medical Psychology, Radboud University Medical Center

[9] Department of Medical Psychology, Maastricht University Medical Center

[10] GGNet, Center for Mental Health, Scelta, Centre of Expertise for Personality Disorders Apeldoorn, PO Box 2003, 7230 GC Warnsveld, the Netherlands

[11] Research Group Arts & Psychomotor Therapies in Health Care, Academy of Health & Vitality, HAN University of Applied Sciences, PO Box 6960, 6503 GL Nijmegen, the Netherlands

*** Correspondence:**
Corresponding Author
blanca.spee@radboudumc.nl, blanca.spee@univie.ac.at

‡ Shared first authorship.

Keywords: Parkinson's disease, Creative Arts Therapy, Transformative Learning, Co-Creation, Critical Neuroscience, Arts-Based Methods, Participatory Action Research, Creativity

Descriptions

D.1 Data gathering

Semi-structured interview guide

In the first round, questions were formulated as open to gain a broader insight into the context. Some of these questions included:

- If you think of someone who is creative, who would it be?
- What is creativity for you?
- Are you creative yourself? What place does creativity have in your life?
- Is there any difference between how you were creative before and after your Parkinson's diagnosis?
- If you think about your childhood, was your life with creativity a bit different or, actually, the same as with Parkinson's?
- Do you do any sport, or do you have any hobbies? Would you consider them as creative?
- Is there a relationship between creativity and dealing with Parkinson's?
- Do you have any expectations in working with an artist and/or creative therapist?

Follow-up questions and group reflections were conducted as fully open questions and focused on having feedback about the intervention sessions, exploring individual and group experiences, and perspective of every participant. In this way, it was possible to implement the study and tailor it for people with Parkinson’s disease.

Tables

Table S1. Themes, cluster, and codes. J, T are coders from the learning sciences and Dutch native speakers, B was a coder from neuropsychology and neuroaesthetics and created codes in English.

Themes	Code Cluster and Codes (Letter in the brackets refer to the coder's initial)
**Anticipation**	
	**Relationship Parkinson's and Creativity**
	Creativity takes away stress (J)
	Creatively busy trains focus (J)
	Parkinson's versus creativity no link (J)
	Parkinson's makes no difference in creativity (T)
	Through creativity/art Parkinson's feels free (J)
	Creativity before or after Parkinson's no different (J)
	Creativity well-being (T)
	Few complaints (T)
	Tired/off mean still enjoyable (T)
	Creative & practical (J)
	Creativity in solid life (J)
	"Agility" (T)
	Looking at the same situation in multiple ways (T)
	Creativity is something you keep doing (T)
	Loneliness connectedness & community (J)
	Leniency agility (J)
	Looking at things in multiple ways (J)
	Looking at things in your own way (J)
	Way of thinking (T)
	"shortcut" (T)
	"Ommetje" is a book of how to experience and handle Parkinson's (T)
	Searching for balance (T)
	Corniness (T)
	Result-oriented (T)
	**Definition creativity**
	Creative follow own feeling (J)
	Creativity definition difficult to give (J)
	Everyone creative in their own way (J)
	Bring yourself in (B)
	Definition creativity (B)
	Taking your emotions into account (B)
	My wife is creative (T)
	Everyone is creative (T)
	Creativity is doing & thinking (T)
	Instrumental creativity (B)
	Athlete is creative (T)
	Different from beaten path (T)

Learning in all areas (T)
Turnaround or Mouthful (T)
Accounting is creative (T)
Creativity is individuality (J)
Shaping things from within yourself (J)
A person is creative (J)
Creativity is new and valuable (J)
Creativity is being busy with art (J)
Creativity is creating/making
Creativity is translation (T)
Creativity is thinking and fun (J)
Creativity is organizing (J)

Imaginativeness

Fantasy (B)
Being creative is a new combination of existing resources (T)
Creative is making new and creating something of value (T)
Reduction of thinking rational
Not so much rational (J)

Motivation & Expectation

To use fantasy (J)
Mental energy (J)
Wanting to develop further (T)
Hope (B)
Personality (J)
Not trying is not knowing (J)
Work makes uncreative, creativity makes you free (J)
Less expectations makes creative (J)
Compelling frameworks (J)
Compelling is alright (T)
Motivation (T)
Status / reputation (B)
Hierarchy is fun (T)
Challenging is important (T)
Practice is compelling you have to go to class (T)
Proceed with creative activities post sessions (B)
Structure vs assumptions (J)
Hidden talents (J)
"Knowing what you can do" (T)
Wishes (B)
Challenged (T)
Going in blank (J)
Expectation very fun (J)
Curiosity (B)
Curious (J)
Freedom and open-mindedness is a condition (T)
Unfolding (T)
Fun (T)

Tangible (T)
Expect out of the box (J)
Go in blank (T)
Curious (T)
I like to be challenged (T)
Keep up with the pace of life (T)
Let me be surprised (T)
Learn a little from the arts (T)

Creative Engagement

Flow & Presence

Forget the time (T)
Focus (B)
Flow + pace (T)
Losing yourself completely in it (J)
Flow (B)
Active (B)
Get lost in structure and rhythm (T)
Structure cadences, rhythm (music, painting) (T)
Flow at a nice pace (J)
Drawing also flows (J)
Cadence almost hypnotizing (J)
Trance nice feeling (J)
Getting lost in it (J)
Rhythm (B)

Fun & Joy

Fun and relaxing (J)
Creation boost (T)
Experience people super (J)
Session is a creative boost (J)
Becoming happy (T)
Fun (B)
Happy (T)
Giving energy (B)
Play (B)
Pleasure (B)
Having fun (T)
Playing is fun (J)
Fun (T)

Open ended

Unexpected outcome of activity (positive) (B)
Expanding (T)
Thinking about, but no end goal (T)
Daydreaming is healthy (J)
Unlimited is nice (J)

Having no purpose but fun (J)
Comfort zone bigger than you thought or me outside it (J)

**Self-expression**
Made curious about inner own creative potential (B)
Self-expression (B)
Presenting myself (T)
Not feeling closed (J)
Tap into creativity (J)
Self-expression (J)
Emotion in body (T)
Letting out (J)
Cry when coming to expression (J)
Expressing (anger) feels good (J)
No plan is nice and tasty (J)
Structure cadence & rhythm music, painting & walking (T)
Crying is wanted (T)
Satisfied? (T)
Emotions release, also crying (T)
Being creative (J)
Materializing (J)
Creative process (B)

**Affective impact of release**
Art moves me (J)
Emotional increase and decrease (T)
Feeling (Y)
Moodier (T)
Loosing fear (B)
Emotion and crying (J)
Amazed (J)
Amazed (T)
Goose bumps (J)
Relaxing (T)
Relaxing (B)

**Autonomy & Freedom**
Freedom (B)
Free (B)
Going through it and then Free (J)
Letting go of control (J)
Jamming (J)
Breaking loose (J)
Getting loose (J)

**Sense of meaning**
Interesting (T)
More precise (T)

Process in yourself (J)
Existential effect of creative activity (B)
Satisfaction (T)
Fulfilling (T)
Non-psychological existential experience from the body (Y)
It is research for looking who you are what can you do (J)

Design

Environment design

Location is pleasant (J)
Not too many participants (J)
Design 10s: more challenging on content & technique (J)
Conviviality with others (J)
Connection (J)
Design 10s: variation important (J)
Medication during the session (T)
Study design issues (B)
Openness (B)
Safety (B)
Connecting (B)
No need to explain condition (B)
No pressure or must (B)
Drop deadline executive tasks (T)
Freedom (T)
Reduced expectations(B)
Need for past association (B)
Diversity (B)
Flexibility in playground vs assignment (B)
Feeling safe (J)
No obligation
Time flies during session (Y)
Space (Y)
Lighting (operation) ("peers") + atmosphere (Y)
Dialogue during session + (J)
Play behavior (Y)
'Code Clusters'!D26
Environment intervention constructing openness, relatedness & safety (J)
Feeling that I know you for a long time (J)
Attention from researchers (J)
Research very carefully (J)
Paradox expecting "blank" vs "structure" (J)
Expectation of intervention (B)
Handle expectation openness? Paradox (J)
Participants need to know what to expect (T)
Depart we on it (T)
Goes very nice further (T)
Getting more clues (J)
Study goal (B)

Shortage of time (T)
No deal (T)
Feeling pleased (T)
No obligation (T)
Casual (T)
Safety (T)
Openness (T)
It is necessarily to try something new (T)
In your body (T)
Giving trying chance (T)

**Intervention: guidance**

Lost standing there (must be possible) (J)
Easier with help (J)
Lock reflection aiming (J)
Guiding is sensing (J)
No creative therapy (J)
What questions do you ask (J)

**Intervention: outcome creative expressions**

Playing with colors (B)
Listening to music (B)
Drawing (B)
Photography (B)
Rough working with the body (B)
Play act theatre play (B)
Preference for one form of theatre (J)
Writing poetry/text (B)
Rough working with colours (B)
Handicraft (B)
Playing music/piano (B)
Filming (B)
Visual (T)
Geometric patterns (T)
Being aware of positive effect of creative activities (B)
Need of spontaneous acts (B)
Wish to participate in activities (B)
Leave comfort zone (B)
Letting go of control (B)
Be open (T)
Wanting to try (T)
"Urgency of this study not to be understood" (T)
Effect Awareness (J)
Changing expectations of outcome (B)
Prolonging functional state (B)
Emotionally more agitated (B)
Heightened emotional control after (B)
Outcome is the learning not to want the final product (T)

	Slow burner: doing is not the result (T)
**Learning & Reflection**	
	**Art as a challenge**
	Tremors during session (T)<br>Challenging (B)<br>In my head I think I can do it (T)<br>Out of comfort zone is fun (T)<br>Not shying away from challenges (T)
	**Adaptivity**
	Experience Flexibility (J)<br>Finding suppleness (T)<br>Problem-solving approach (T)<br>Rethinking (J)<br>Coping (B)<br>Develop other tactics (J)
	**Problem-solving**
	Creativity is problem-solving approach (J)<br>Finding solutions for issues (B)<br>Solving practical things is creative (T)<br>Creative solution to treatments (T)
	**Learning & reflection**
	Examine yourself/investigate yourself (J)<br>Change prospective (B)<br>Great lesson for myself (learning from other participants) (J)<br>Discovering yourself surprised (J)<br>"I can't do that" (J)<br>Creativity is developable (J)<br>I'm not good at x (J)<br>Learning must come from myself (J)<br>Growth mindset (with limits) (J)<br>Finding balance is learning (T)<br>Perceptive (B)<br>Big halls and TV camera is good learning (T)<br>"Activity is good for me" self-reflection needs others (J)<br>Disoriented (T)<br>Getting through it (T)<br>Going deep (peeling off & barricades through) (T)<br>Stand optimised / further complete / even more beautiful (T)<br>No creative art in youth (J)<br>I am good at reflecting (T)<br>Am I good at drawing (T)<br>Difficult to create things myself (T)<br>Over the threshold (J)

Not in the mood for something new (J)
Trying helps (T)
Language vs level reflection (J)
Giving words (J)
Not wanting to be perfect (J)
Trying things (J)
Doing things, yourself (J)
Tactile (T)
Going deep (T)
Stepping out of frame "doesn't necessarily have to" (T)

Acceptance is a creative process

Don't get depressed (T)
Not rebellious vs Parkinson's (T)
Choice what fits (T)
Acceptance (B)
Admitting failure (J)
Giving up old life (J)
Acceptance itself as a creative process (J)
Prolonged process of acceptance (T)
Problem of acceptance lies with me (T)
Changing goals in life (T)
Not seeing Parkinson's as a disease (T)
Staying positive (B)
Practice letting things go (J)
Reasoning does not help (J)
Who am I inclusive (T)

Living with Parkinson's

Hobbies

Art & literature (T)
Basketball
Texts (T)
Textile crafting (T)
Theatre has interest but not at the forefront (T)
Theatre (T)
Collage (T)
Handicrafts (T)
Cycling (T)
Walking (T)
Digital visual working (T)
Gardening (T)
Collect (T)
E-bike is a wonderful intervention (T)
Studying (T)
Jeux de Boules (T)

Hobby (B)
Many sports (J)
Physical activities (B)
Football with people with Parkinson’s (T)
Music (T)
Physiotherapy (T)

**Living with Parkinson’s disease**

Being trapped (dip) (T)
Energy swing (J)
Caught in energy swing (J)
Writing, I would rather not do anymore (overview, stiff) (T)
Daily life ripples (J)
Being scared (T)
Day with Parkinson’s (B)
Tired in the evening (J)
Lucky no energy dips (J)
Unable to let go is addictive behaviour pre-diagnosis or perfectionism (T)
Don't think, just do (J)
Focus is on Parkinson's and that is difficult (T)
Not working anymore is also nice (T)
Equals/peers (T)
Associated creative people (B)
Environment must also accept (J)
Few people know what Parkinson's is (T)
Social surrounding (B)
Stigma (T)
People quality of live is less because of Parkinson’s (T)
Tolerance (T)
Colleagues respond well (T)
Students take notice (T)
Other person's perspective (T)
Too busy (people mass) (T)
Finding it difficult (T)
Old group is confrontative and stressful (T)
"Hospitalization" (T)
"knowing who is there" (T)
"Knowing the place" (T)
Docent/therapist wetter deal with Parkinson's (T)
Companionship is important (sociable, social is stick) (T)
Location is important (T)
Serendipity: "you must have" (T)
Because of Parkinson’s I don't have to just run with it (T)
Control of voice Gone because of Parkinson's? (T)
Stiffness (T)
It sucks that disease (T)
Parkinson's à hurt (T)
Parkinson's flattens out emotions (T)
Free day care must Because of PD (T)

Suffer from Freezing (T)
No energy dips (T)
Hard to hep (T)
Becoming passive because of Parkinson’s (T)
"Despite Parkinson’s" (T)
From home (T)
Difficulty choosing (J)
Plate sticks (J)
Thinking falters (J)
Parkinson’s feels like a rubber band around me (J)
Reluctant in normal life (J)
Consequence Parkinson’s (B)
Blame Parkinson’s (B)
Constant evaluation (B)
Control time (dopamine level) (B)
Relatives: why do you still have to do that? You're sick, aren't you (T)
Difficulty in making plans (B)
Increased vulnerability due to PD (B)
Difficulty writing (B)
Survival (J)
Emotional flattening (B)
Thrown back on life (T)
Parkinson's symptoms (B)
Fear of increased symptoms (B)
Feeling stuck (B)
New time after work (T)
Giving lectures is also theatre (T)
Not in the spotlight (T)
Shy (J)
Not afraid to be quizzable (J)
Not in the spotlight (T)

Dopamine

Prodromal parkinsonian symptoms (B)
DBS (J)
Parkinson's effect on creativity (B)
Reduce binary thinking (J)
Therapies (B)
Dopamine is happiness hormone (T)
Dopamine (T)
Medicines (T)
DBS surgery (T)
Freezing & medication (J)
Vibration & emotion (J)
Medical care (B)
OFF...but still fun (J)
Dealing with low dopamine (T)
Effect dopamine (B)
The patient's dopamine frame (J)

Dopa - feeling restless (J)
Relationship Parkinson’s and creativity is negative (J)
Forgetting medication during session (J)

**Getting older with Parkinson's**

Getting older less insecure (J)
Matter of age (B)
Parkinson's ? You also got older!
Older is less insecure (T)
Thinking of the past (B)
Remembering (B)

**Past**

Family from the past(J)
Stories from the past (J)
Memories (J)
Back when I was a kid (J)
Played guitar (J)
No more singing Parkinson’s(J)
Pre-existing creative activities (B)
Occupational experience (B)
Creative education (B)
Knitting crochet quilting (T)
Clothing & fabric working (T)
Last time painting was 2011 (T)
Used to do many other sports (T)
Played guitar (no longer goes) (T)
Childhood (B)
Past hobbies (B)
Gone away (J)
Drawing lessons before (J)
As a child (J)
Organ lessons before (J)
Associated creative activities (B)

**PD_Peers**

Shared suffering (T)
Connecting with people (T)
Caregivers understand Parkinson's (T)
Vulnerable moment (J)
Privately Parkinson's is magnified (T)
Too vulnerable in your life (T)

**Self-worthiness**

Who am I (B)
Individuality (T)
Comprehensiveness (T)
Take part (J)

Real participation (Y)
Recognition (J)
Do I still contribute with Parkinson's (T)
I am a bit of a go-getter (T)
Making a difference (J)
