# Supplementary material for: Co-creating a person-centered creative engagement intervention for Parkinson's care
Source: Front Psychol. 2025 Jan 15;15:1469120. doi: 10.3389/fpsyg.2024.1469120 (PMC11774897; doi:10.3389/fpsyg.2024.1469120)
Supplement: Supplementary file 2 [file Supplementary_file_1.pdf]

Co-Creating a Person-Centered Creative Engagement Intervention for Parkinson's Care

Blanca T.M. Spee[1,2,3,*‡], Thieme B. Stap[4,5,‡], Marjoke Plijnaer[6], Gert Pasman[7], Sara Zeggio[1], Annelien Duits[8,9], Julia S. Crone[2], Suzanne Haeyen[10,11], Matthew Pelowski[2,3], Bastiaan R. Bloem[1], Jan-Jurjen Koksma[4]

[1] Radboud university medical center, Donders Institute for Brain, Cognition and Behaviour; Department of Neurology, Centre of Expertise for Parkinson & Movement Disorders, Nijmegen, the Netherlands

[2] Vienna Cognitive Science Hub, University of Vienna, Vienna, Austria

[3] Department of Cognition, Emotion, and Methods in Psychology, Faculty of Psychology, University of Vienna, Vienna, Austria

[4] Radboud university medical center Health Academy, Nijmegen, the Netherlands

[5] Fontys University of Applied Sciences, Research Group Professional Workplaces, Eindhoven, The Netherlands

[6] Art Unbound, collaboration partner of Radboud university medical center, Nijmegen, the Netherlands

[7] Faculty of Industrial Design Engineering, Delft University of Technology, Delft, the Netherlands

[8] Department of Medical Psychology, Radboud University Medical Center

[9] Department of Medical Psychology, Maastricht University Medical Center

[10] GGNet, Center for Mental Health, Scelta, Centre of Expertise for Personality Disorders Apeldoorn, PO Box 2003, 7230 GC Warnsveld, the Netherlands

[11] Research Group Arts & Psychomotor Therapies in Health Care, Academy of Health & Vitality, HAN University of Applied Sciences, PO Box 6960, 6503 GL Nijmegen, the Netherlands

*** Correspondence:**
Corresponding Author
blanca.spee@radboudumc.nl, blanca.spee@univie.ac.at

‡ Shared first authorship.

Keywords: Parkinson's disease, Creative Arts Therapy, Transformative Learning, Co-Creation, Critical Neuroscience, Arts-Based Methods, Participatory Action Research, Creativity

Appendix A. Impressions of first iteration

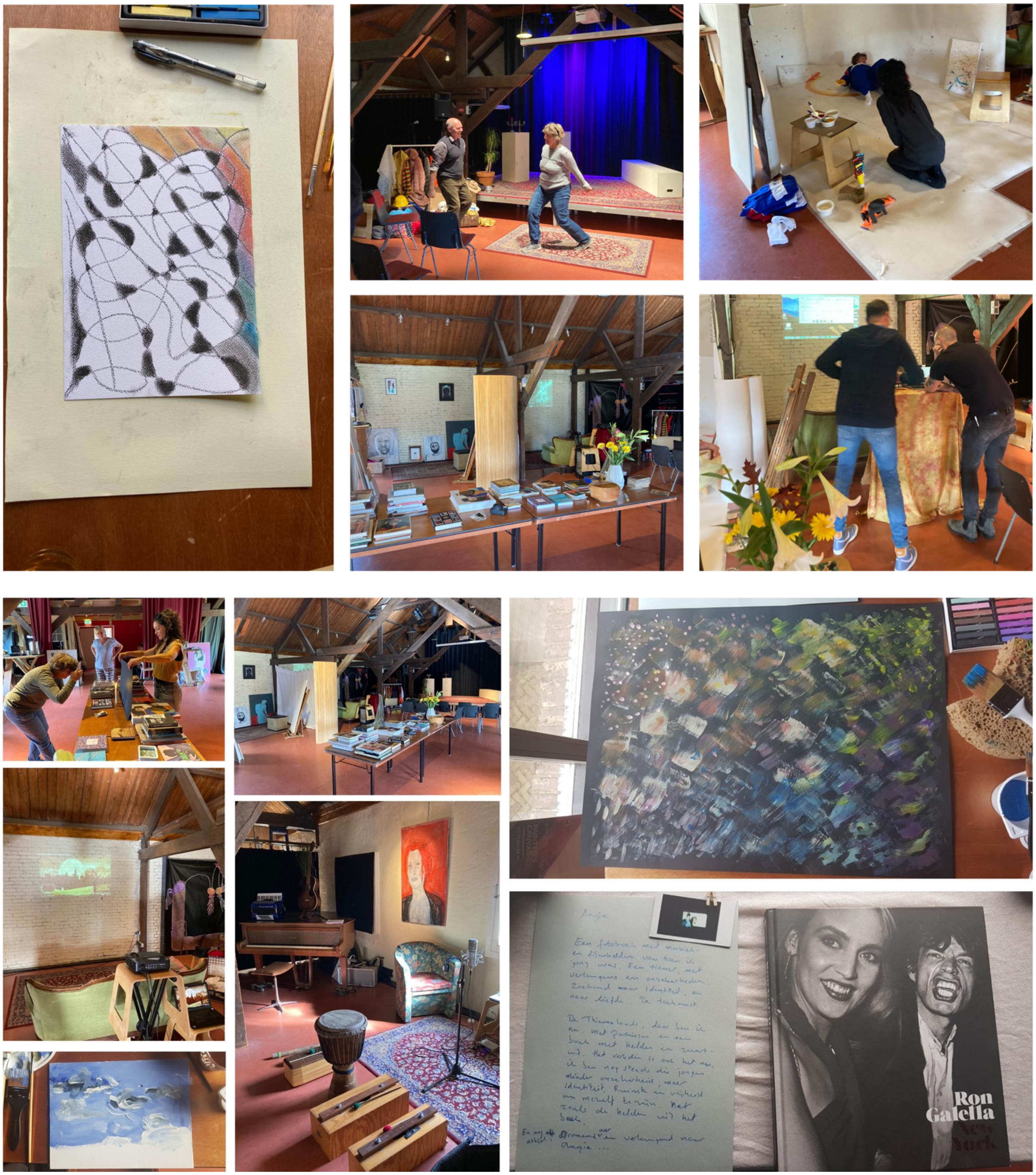

Appendix B. Impressions of second iteration

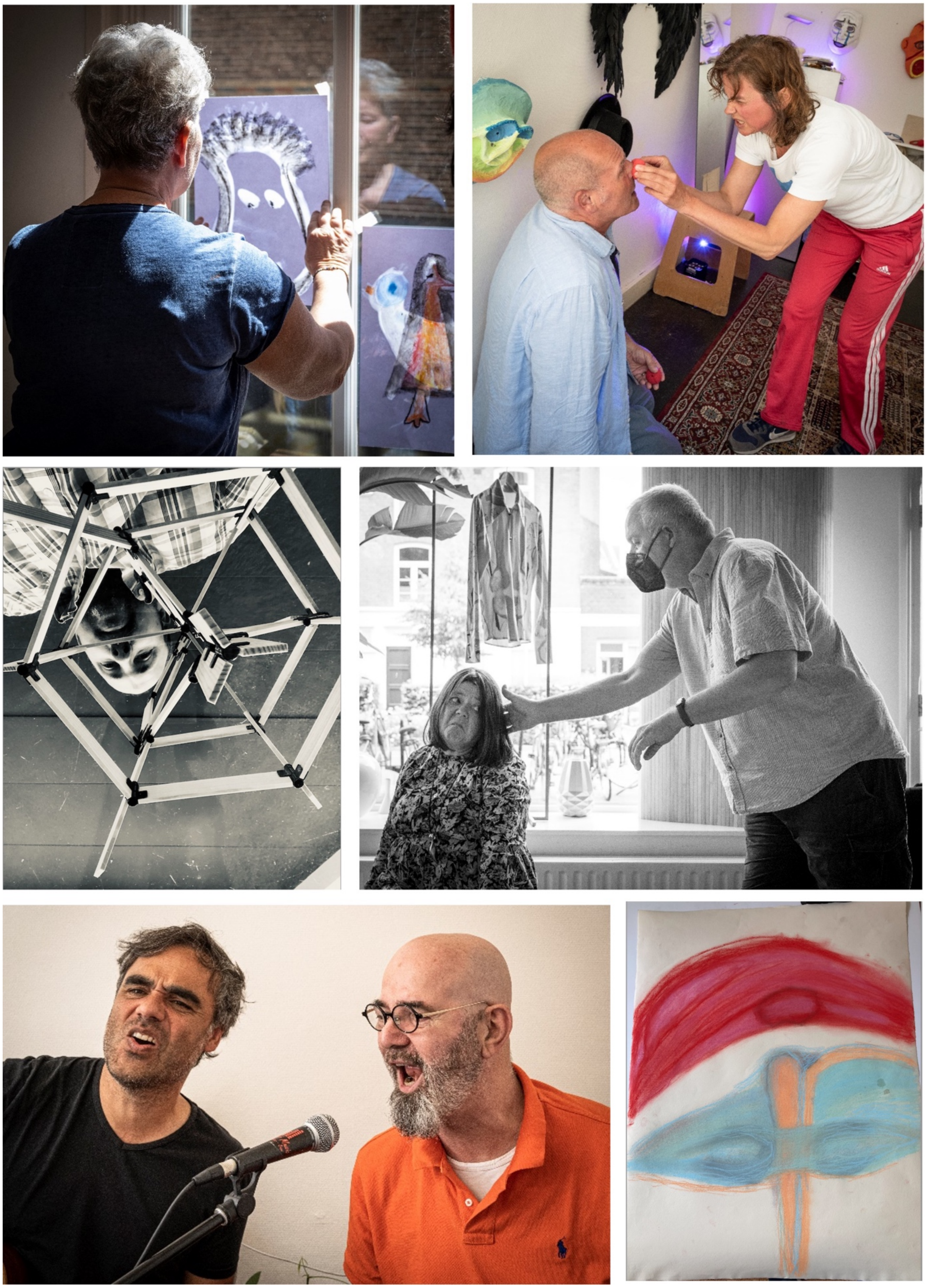

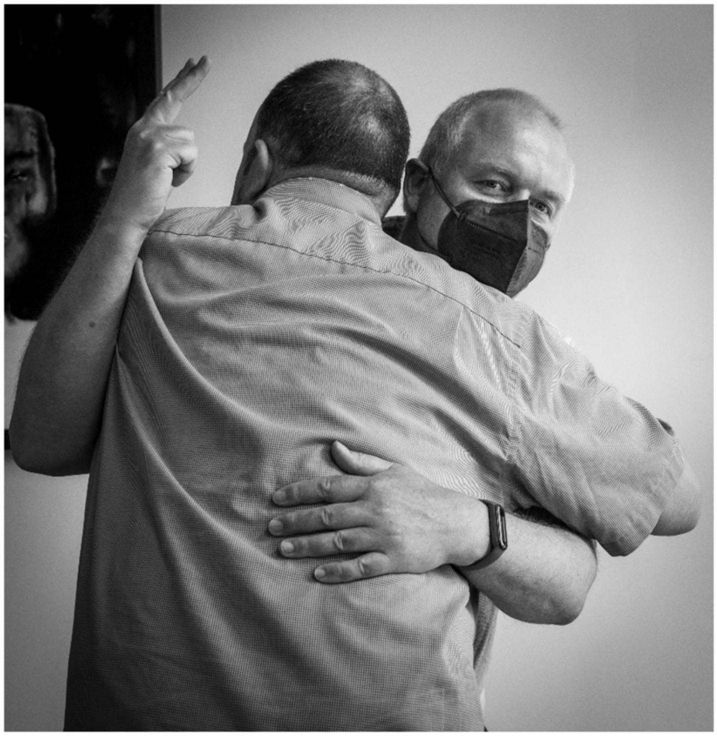

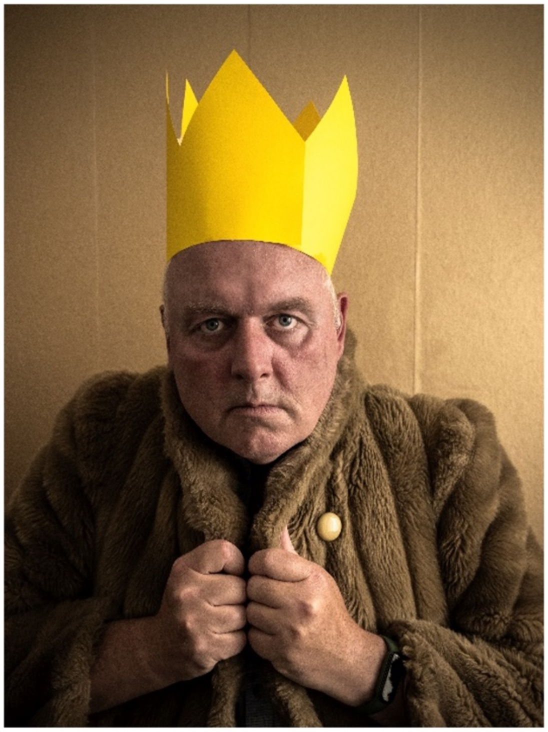

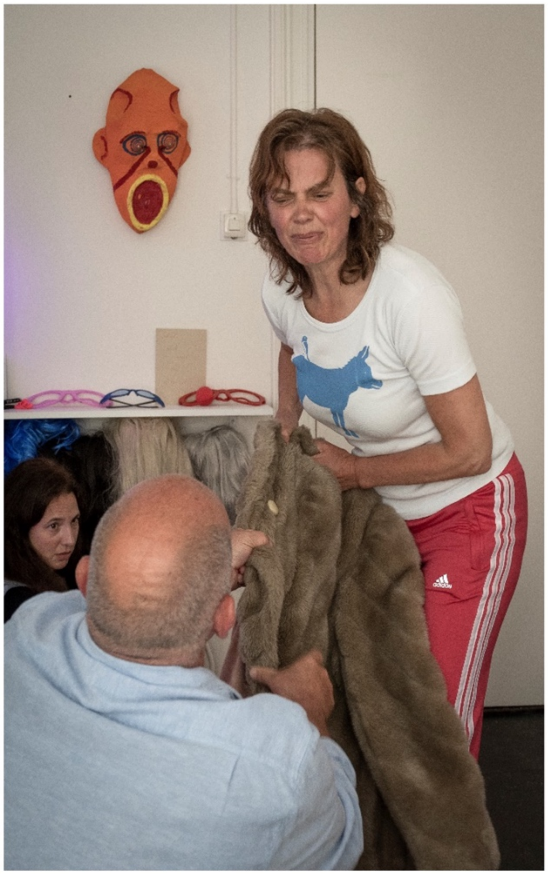

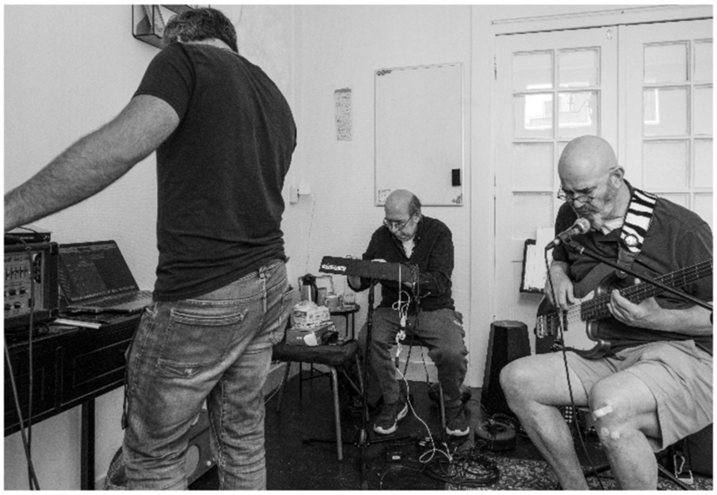

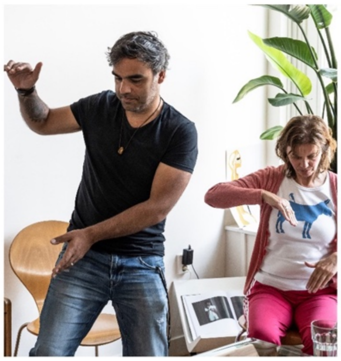

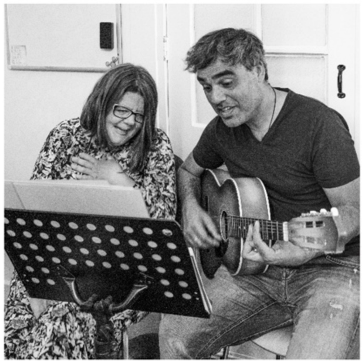

Appendix C. Poem of a person with Parkinson’s in Dutch

Box B. A poem written by a participant (Dutch version).

“De eindigheid van tijd”

Genieten van het kleine moment,
Hier en nu, maar heel content.

Even niet dat doolhof van gedachten,
Wat staat mij nog te wachten?

Soms door de bomen het bos niet meer zien,
Toch geef ik het leven nog een tien.

Dan overweldigd, vaak verwonderd,
Hoe groots de ruimte om ons heen, overdonderd.

Wat is mijn plek? Wat is mijn doel?
Wat breng ik teweeg? Wat is het dat ik voel?

Wat blijft er over als ik klaar ben?
Ik weet het niet, het onbekende dat ik nog niet ken.

Weer een stap terug, en dan toch spijt,
Niet meer kunnen doen, wat je toen nog wel kon, wel kon in die tijd.

Maar tijd gaat voorbij, zonder te wachten,
Op die trage man, waarmee we vroeger lachten.

Zijn tijd is eindig, dat is niet uniek,
Maar hij kan steeds minder, was ik maar niet ziek.

Geniet van het kleine moment,
Hier en nu, maar heel content.
